# Metabolic Engineering of *E. coli* for Enhanced Diols Production from Acetate

**DOI:** 10.1021/acssynbio.4c00839

**Published:** 2025-03-19

**Authors:** Luca Ricci, Xuecong Cen, Yuexuan Zu, Giacomo Antonicelli, Zhen Chen, Debora Fino, Fabrizio C. Pirri, Gregory Stephanopoulos, Benjamin M. Woolston, Angela Re

**Affiliations:** † Department of Chemical Engineering, Massachusetts Institute of Technology, Cambridge, Massachusetts 02142, United States; ‡ Centre for Sustainable Future Technologies, Fondazione Istituto Italiano di Tecnologia, Via Livorno 60, 10144 Turin, Italy; § Department of Applied Science and Technology, Politecnico di Torino, Corso Duca degli Abruzzi 24, 10129 Turin, Italy; ∥ RINA Consulting S.p.A., Energy Innovation Strategic Centre, Via Antonio Cecchi, 6, 16129 Genoa, Italy; ⊥ Department of Chemical Engineering, Massachusetts Institute of Technology, Cambridge, Massachusetts 02142, United States; # Department of Chemical Engineering, Key Laboratory of Industrial Biocatalysis (Ministry of Education), Tsinghua University, Beijing 100084, China; ∇ Department of Environment, Land and Infrastructure Engineering, Politecnico di Torino, Corso Duca degli Abruzzi 24, 10129 Turin, Italy; ○ Department of Chemical Engineering, Northeastern University, 360 Huntington Avenue, 223 Cullinane, Boston, Massachusetts 02115, United States

**Keywords:** synthetic biology, sustainability, acetate valorization, diol, gas fermentation

## Abstract

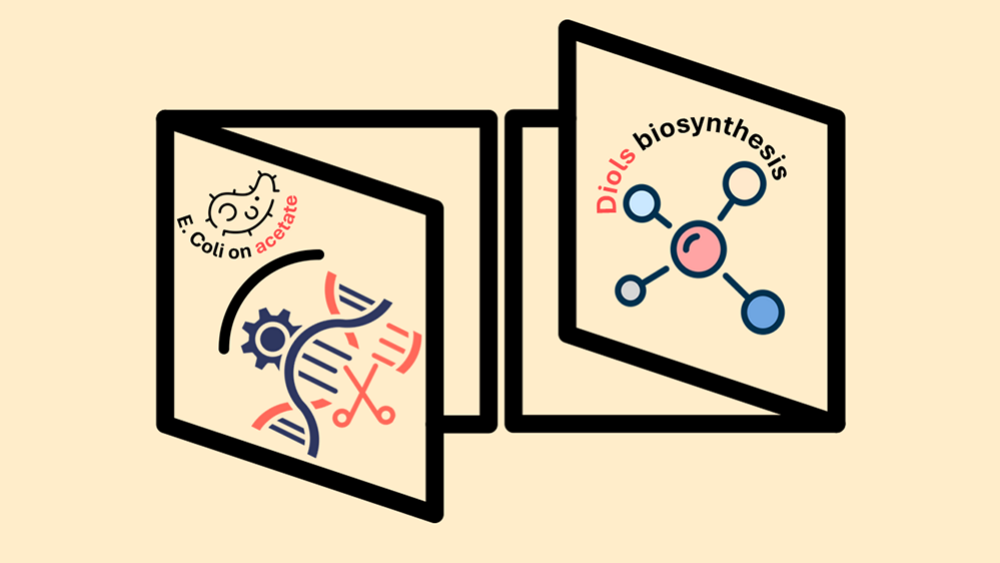

Effective employment of renewable carbon sources is highly demanded to develop sustainable biobased manufacturing. Here, we developed *Escherichia coli* strains to produce 2,3-butanediol and acetoin (collectively referred to as diols) using acetate as the sole carbon source by stepwise metabolic engineering. When tested in fed-batch experiments, the strain overexpressing the entire acetate utilization pathway was found to consume acetate at a 15% faster rate (0.78 ± 0.05 g/g/h) and to produce a 35% higher diol titer (1.16 ± 0.01 g/L) than the baseline diols-producing strain. Moreover, singularly overexpressing the genes encoding alternative acetate uptake pathways as well as alternative isoforms of genes in the malate-to-pyruvate pathway unveiled that leveraging *ackA-pta* and *maeA* is more effective in enhancing acetate consumption and diols production, compared to *acs* and *maeB*. Finally, the increased substrate consumption rate and diol production obtained in flask-based experiments were confirmed in bench-scale bioreactors operated in fed-batch mode. Consequently, the highest titer of 1.56 g/L achieved in this configuration increased by over 30% compared to the only other similar effort carried out so far.

## 1 Introduction

Major strides have been made in the biobased production of highly diverse and valuable chemicals that are acutely needed for sustainable civilization.^1−3^ However, the common carbon sources employed in industrial fermentation processes are obtained from feedstocks that compete with the food and feed industries.^4,5^ Against this backdrop, acetate is an attractive alternative microbial carbon source for several reasons including mainly its production in robustly effective amounts by both biological and chemical means at lower costs compared to conventional carbon feedstocks.^6^ While chemical processes such as methanol carbonylation account for most of the acetate production,^7−10^ the biotechnological approaches include lignocellulosic biomass refinery through chemical, enzymatic, and thermal technologies,^11^ anaerobic fermentation of single-carbon (C_1_) gases using acetogenic bacteria,^12,13^ and, albeit to a lesser extent, microbial electrosynthesis.^14,15^

Acetate can be activated to acetyl-Coenzyme A (acetyl-CoA) and assimilated to glyoxylate/tricarboxylic acid (TCA) cycle intermediates, which are the starting precursors to produce value-added compounds in most industrially relevant microorganisms.^16,17^ Along with natural compounds, such as lipids,^18^ several synthetic metabolic pathways^19^ have extended the assortment of acetate-based chemicals such as acetone,^20^ isopropanol,^21^ succinate,^22^ and polyhydroxyalkanoate,^23^ in addition to many more.^24^ Furthermore, the integration of acetate-producing with acetate-consuming fermentation processes is emerging as a promising paradigm to ultimately yield energy-intense and high-value biobased products.^6,24−27^

**Figure 1:**
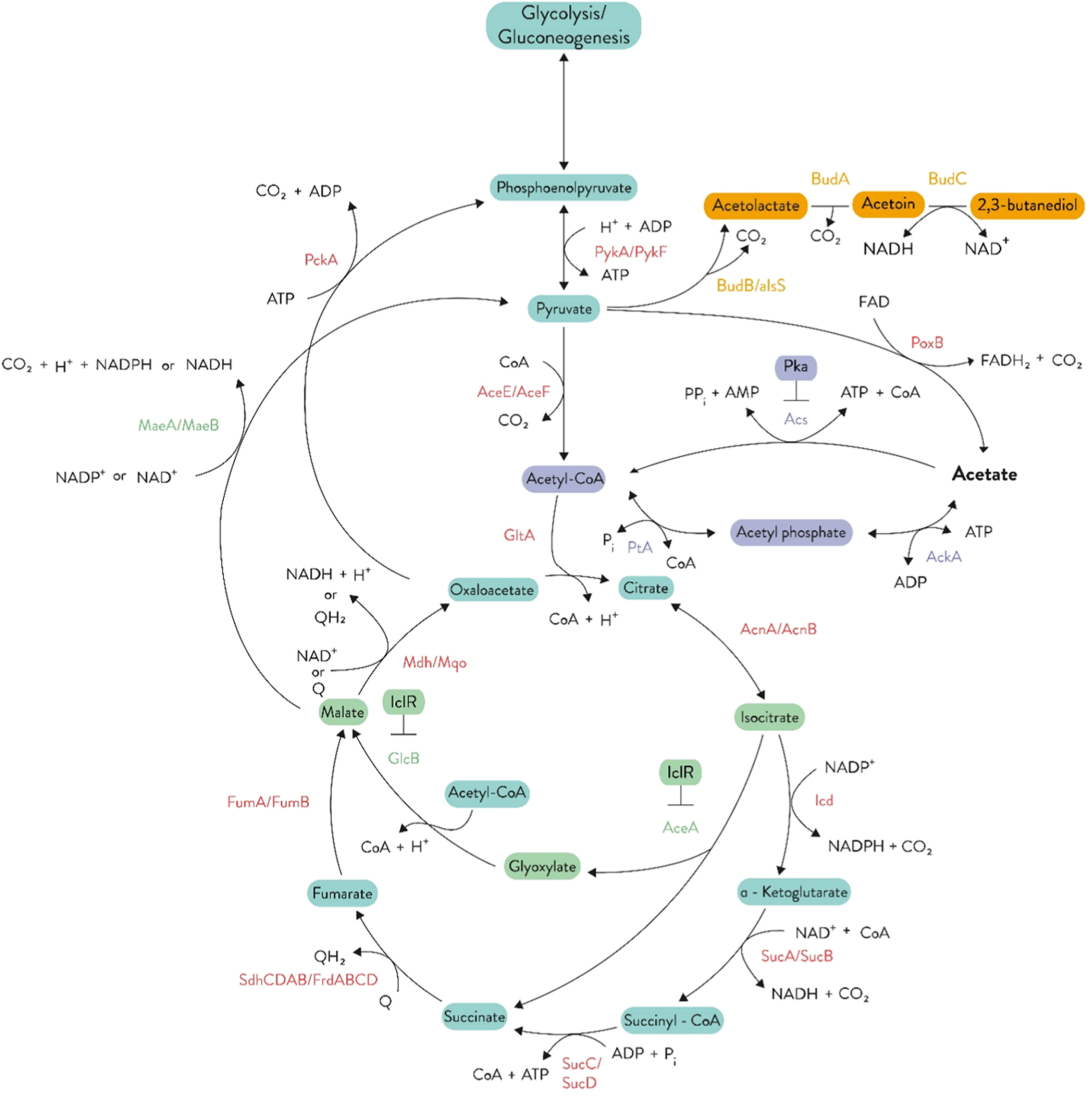
Graphical representation of the acetate metabolism in *E. coli*. The figure reports the main enzymes of the pathways (light red), central intermediates, cofactors, and final products. The figure also shows the heterologously expressed pathway for acetoin and 2,3-butanediol production. Abbreviation: AckA, acetate kinase; Pta, phosphotransacetylase; Acs, acetyl-CoA synthetase; GltA, citrate synthase; AcnA/AcnB, aconitate hydratase; Icd, isocitrate dehydrogenase; SucA/SucB, α-ketoglutarate dehydrogenase; SucC/SucD, succinyl-COA synthetase; SdhCDAB/FrdABCD, succinate dehydrogenase/fumarate reductase; FumA/FumB, fumarate hydratase; Mdh/Mqo, malate dehydrogenase; AceA, isocitrate lyase; GlcB, malate synthase; MaeA/MaeB, malate dehydrogenase; PckA, phosphoenolpyruvate carboxykinase; PykA/PykF, pyruvate kinase; AceE/AceF, pyruvate dehydrogenase; PoxB, pyruvate dehydrogenase; BudB/alsS, acetolactate synthase; BudA, acetolactate decarboxylase; BudC, 2,3-BDO dehydrogenase; FADH_2_, flavin adenine dinucleotide dihydrogen; FAD, flavin adenine dinucleotide; NAD(P)H, nicotinamide adenine dinucleotide (phosphate) hydrogen; NAD(P)^+^, nicotinamide adenine dinucleotide (phosphate); CO_2_, carbon dioxide; CoA, coenzyme A; ATP, adenosine triphosphate; ADP + P_i_, adenosine diphosphate + phosphate; [H], reducing equivalents. Metabolites and enzymes involved in the diol production pathway, the acetate assimilation pathway, and the glyoxylate shunt are highlighted and reported in orange, violet, and green, respectively. The Pka and IclR repressors are displayed at the represented regulated genes. This image was adapted from.^51^

This study developed *Escherichia coli* strains for 3-hydroxybutanone (acetoin) and 2,3-butanediol (2,3-BDO) production using acetate as the sole carbon substrate. 2,3-BDO has a considerable range of applications, including both its direct use in manufacturing personal care products, food additives and flavorings, antifreeze agents, plant growth promoting agents,^28,29^ and its indirect use by further transformation to value-added products such as 1,3-butadiene and methyl ethyl ketone (MEK).^30,31^ Acetoin is widely used as a building block for the synthesis of various chemicals, cosmetics compounds, and pharmaceuticals and as a flavoring enhancer in the food industry owing to its buttery taste.^30,32,33^ The biosynthetic routes of acetoin and 2,3-BDO are highly intertwined, as acetoin is the immediate precursor of 2,3-BDO, Indeed, they are formed from pyruvate in few steps: first, an acetolactate synthase (BudB/alsS) links two molecules of pyruvate to α-acetolactate; second, an acetolactate decarboxylase (BudA) splits acetolactate into acetoin and CO_2_; third, a 2,3-BDO-dehydrogenase (BudC) reduces acetoin to 2,3-BDO. 2,3-BDO can be reversibly transformed into acetoin to regenerate NADH to preserve a constant oxidation–reduction state (Figure 1). Because the bioproduction of acetoin and 2,3-BDO leans on common enzymatic reactions ultimately leading to diol formation, 2,3-BDO and acetoin are here referred to as diols, in agreement with previous studies.^34^

Our study developed and assessed several metabolically engineered *E. coli* strains for efficiently supplying acetyl-CoA from acetate and parallelly promoting acetate-based diols production. The acetate uptake and diol production in *E. coli* have been individually investigated in several previous studies over the past years. Many examples describe *E. coli* engineering for 2,3-BDO^35−41^ and acetoin^42−45^ heterologous production. However, these studies do not overlap except marginally with our study, as they mainly focus on the utilization of sugars rather than acetate as carbon substrate. Conversely, several metabolic engineering interventions, including some we shall recall hereafter, are suited to enhance acetate activation and assimilation in *E. coli*, yet their effects on diols production are unknown. *E. coli* possesses two main routes for acetate activation to acetyl-CoA (Figure 1). Following uptake, acetate can be activated to acetyl-CoA either by the low-affinity *ackA-pta* pathway,^46^ which is preferentially active when acetate is present at high concentration, or by the high-affinity *acs* pathway,^47^ which is expected to be active at low concentrations.^48−50^ The first route is operated by acetate kinase (AckA) and phosphotransacetylase (Pta), which leads to ADP and acetyl-CoA generation. The second route consists of the irreversible reaction catalyzed by the acetyl-CoA synthetase (Acs), which leads to AMP and acetyl-CoA generation.^48,51,52^ The two pathways differ in energy demand as one ATP mole is consumed in the AckA-Pta pathway while the pathway mediated by Acs requires two ATP moles.^53^ Both pathways lead to the formation of acetyl-CoA that can subsequently enter the TCA cycle, which acts as a terminal pathway of respiration and provides obligatory precursors of the macromolecular constituents of the cell (Figure 1). When cells are grown on acetate, cells increase the flux toward the glyoxylate shunt, which is composed of an isocitrate lyase (AceA), which catalyzes the aldol cleavage of isocitrate to succinate and glyoxylate, and a malate synthase (GlcB), which catalyzes the synthesis of malate from glyoxylate and acetyl-CoA.^54^

Metabolic engineering interventions acting on the *ackA-pta*(20,55−57) or the *acs* pathway^58−60^ were proven to improve acetate assimilation and utilization. Other studies impinged on the transcriptional or post-translational regulation of genes in the glyoxylate shunt and acetate uptake. For instance, the deletion of *iclR*, which transcriptionally represses the *aceBAK* operon, and the deletion of the peptidyl-lysine N-acetyltransferase encoded by *pka*, which acetylates Acs, were found to favor acetate assimilation into biomass and bioproducts.^51,61^

Only one attempt was carried out to produce diols from acetate in *E. coli*. This study developed an *E. coli* W strain expressing a construct with 2,3-BDO biosynthetic genes (*E. coli* W 445_Ediss) and an *E. coli* W strain (445_Ediss Δ4) that differs from it for the deletion of genes responsible for the formation of the mixed acid fermentation products.^37^ However, the mutant *E. coli* strains developed in ref (37) were not meant to promote the cultivation of *E. coli* on acetate as carbon source. The present study aimed at developing an *E. coli* strain that can produce diols while growing satisfyingly on acetate. To this end, we introduced a heterologous pathway synthesizing diols in *E. coli*, and we leveraged our knowledge of acetate assimilation and utilization pathways and of their regulatory features to further improve diols production. Intervening on the acetate uptake and malate-to-pyruvate conversion was found to be effective in increasing the metabolic flux toward diols. Of note, the diols production increments, obtained in flask-based experiments, were confirmed using bench-scale bioreactors operating in fed-batch configuration. Indeed, the titer of 1.56 g/L of diols surpassed the most promising titer achieved so far by 30%. This study supported the potential of acetate as a sustainable substrate for bioproduction and laid the basis for further developing appropriate metabolic engineering approaches in *E. coli* to profitably enhance acetate transformation into desired products such as diols.

## 2 Results and Discussion

### 2.1 Development of Diols-Producing *E. coli* Strains and Testing Using either Acetate or Glucose

We started our investigation with the selection of the *E. coli* strain to be employed as the biocatalyst for the conversion of acetate into 2,3-BDO and acetoin. Both *E. coli* W (ATCC 9637) (hereafter *E. coli* W) and *E. coli* BL21 (DE3) (hereafter *E. coli* BL21) were considered, as each of them has displayed favorable traits. Indeed, both strains are highly tolerant toward acetate. The acetyl-CoA synthetase and the glyoxylate shunt were shown more active in *E. coli* BL21 than in *E. coli* W.^62^ On the other side, *E. coli* W was the host strain elected for the sole attempt reported so far to produce 2,3-BDO and acetoin from acetate.^34,37^

#### 2.1.1 Heterologous Assembly of the Diols Biosynthetic Pathway Is Not Affected by Gene Order or Plasmid Copy Number

The *budA*, *budB* and *budC* genes from *Enterobacter cloacae* subsp. dissolvens were selected as donor genes to assemble the 2,3-BDO biosynthetic pathway in *E. coli* by relying on the results of previous attempts to develop *E. coli* strains for the microbial 2,3-BDO production.^37^ Furthermore, since the heterologous expression of multiple enzymes can be influenced by the plasmid copy number and by the gene order within the operon, we comparatively assessed p5T7 and pETDuet1 as, respectively, low- and high-copy number plasmids, and two different gene orders, the “*budBAC*” order reflecting the occurrence of the enzymes in the metabolic pathway, i.e., *budB-budA-budC*, and the reverse order, “*budCAB*”. Consequently, four different *E. coli* strains (Table 1) were developed and tested for their ability to produce diols.

**Table 1:** Description of Strains Created during the Development of Diols-Producing *E. coli* Strains and Testing on either Acetate or Glucose^a^

strain name	description
*E. coli* BL21 (DE3)_p5T7_*budCAB*	*E. coli* BL21 (DE3) strain carrying the p5T7 plasmid with the *budC*, *budA* and *budB* gene order
*E. coli* BL21 (DE3)_p5T7_*budBAC*	*E. coli* BL21 (DE3) strain carrying the p5T7 plasmid with the *budB*, *budA* and *budC* gene order
*E. coli* BL21 (DE3)_pET_*budBAC*	*E. coli* BL21 (DE3) strain carrying the pET plasmid with the *budB*, *budA* and *budC* gene order
*E. coli* W_pET_*budBAC*	*E. coli* W (ATCC 9637) strain carrying the pET plasmid with the *budB*, *budA* and *budC* gene order

a It is worth noting that the pET and pCDF plasmids used were a modified version of the original plasmids. Specifically, the T7 promoter was replaced by the Tac promoter.

All four strains were able to produce 2,3-BDO and acetoin from glucose, with 5.94 ± 0.28, 5.91 ± 0.03, 6.39 ± 0.06, and 6.48 ± 0.01 g/L of diols corresponding to *E. coli* BL21 (DE3) p5T7_*budCAB*, *E. coli* BL21 (DE3)_p5T7_*budBAC*, *E. coli* BL21 (DE3)_pET_*budBAC* and *E. coli* W_pET_*budBAC*, respectively (Figure S1). Therefore, we did not gather evidence to support the possibility that the choice of the strain, the gene order, or the plasmid copy number in the biosynthetic pathway influence diols production from glucose.

#### 2.1.2 *E. coli* W_pET_*budBAC* Is Suitable for Engineering the Acetate-Based Diols Production

**Figure 2:**
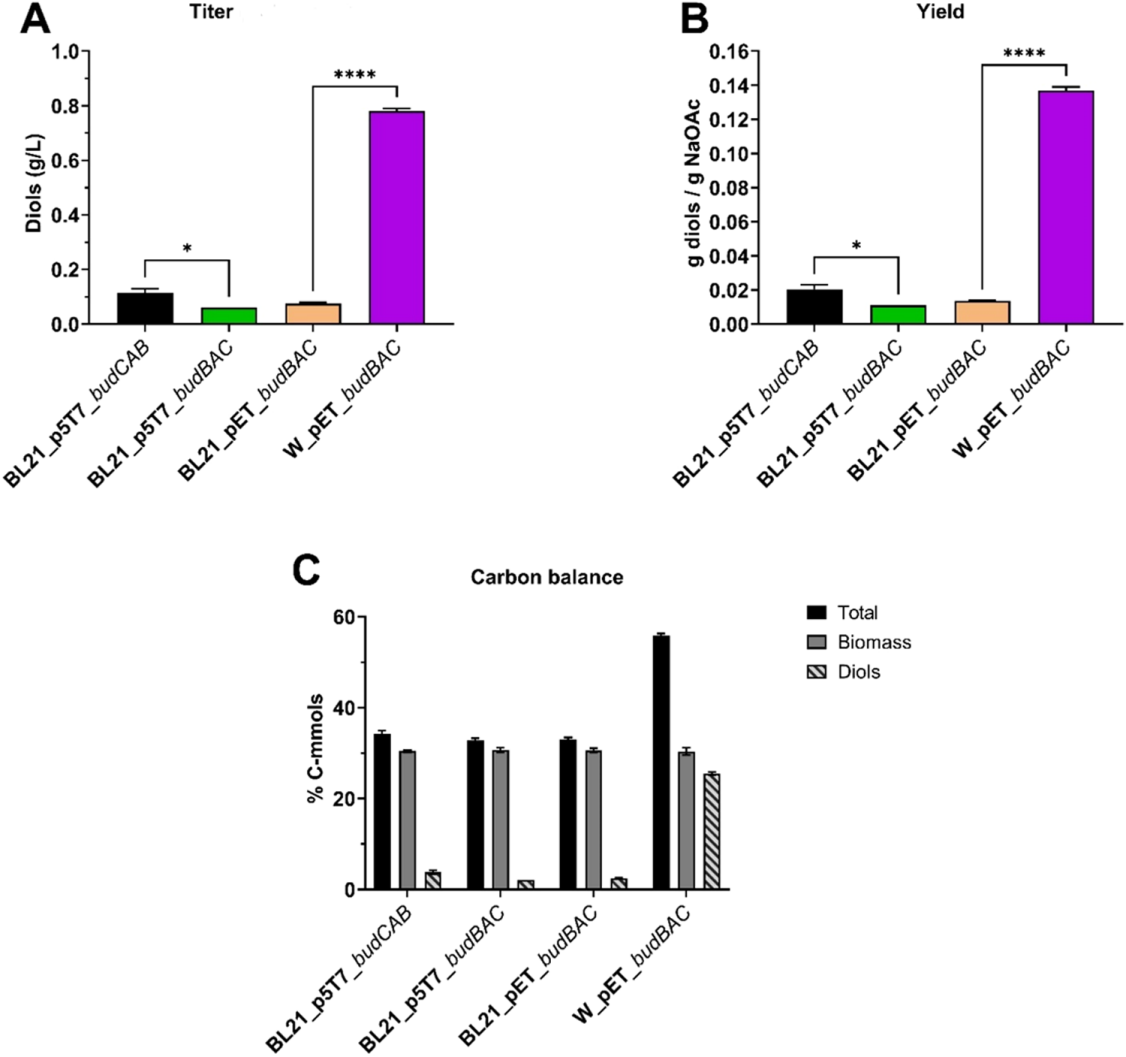
Total diols concentrations, maximum diols yields, and carbon balance analysis of the four *E. coli* strains developed, cultivated in shake flasks with a chemically defined medium containing 5 g/L of sodium acetate. Investigated strains included: *E. coli* BL21 (DE3)_p5T7_budCAB (black), *E. coli* BL21 (DE3)_p5T7_budBAC (green), *E. coli* BL21 (DE3)_pET_budBAC (light orange), and *E. coli* W_pET_budBAC (purple). (A) Total diols (2,3-BDO and acetoin) maximum titers. (B) Total diols maximum yields (g/g). (C) Carbon balance analysis as a molar percentage of the carbon supplied in the products (details in Table 5). Total carbon is considered as the sum of the carbon transformed into biomass and diols. It varied across the four strains, since *E. coli* W_pET_budBAC surpassed the remaining *E. coli* strains in diols production. The average of two independent replicates is plotted for each tested condition. Error bars indicate the standard error of the mean (SEM). Tukey’s test P: *P < 0.0332 and ****P < 0.0001.

Diols production of the four strains was tested in a chemically defined medium supplemented with 5 g/L of sodium acetate (Figure 2). Analyzing diols titer, yield and carbon balance consistently pointed at *E. coli* W strain as the baseline strain for further strain engineering developments. More precisely, *E. coli* W_pET_budBAC produced diols at titers that were 6.88-, 12.54- and 10.24-fold higher than those in *E. coli* BL21 (DE3) p5T7_budCAB, *E. coli* BL21 (DE3)_p5T7_budBAC and *E. coli* BL21 (DE3)_pET_budBAC, respectively (Figure 2A). Diols yield and productivity for *E. coli* W_pET_*budBAC* were found to be equal to 0.14 ± 0.00 g/g and 0.04 ± 0.00 g/L/h, respectively (Figure 2B). Furthermore, *E. coli* W_pET_*budBAC* routed 25.50 ± 0.39% of the supplied carbon (Cmmol) into diols, whereas this percentage does not exceed 2–4% in the remaining strains (Figure 2C). Of note, 2,3-BDO production was transient as 0.24 ± 0.01 g/L of 2,3-BDO were produced in 11 h but were suddenly converted back into acetoin in 24 h (data not shown). This phenomenon is likely to occur since 2,3-BDO conversion to acetoin allows *E. coli* to gain NADH of which it experiences a shortage in the final stage of growth.^34^ It is worth mentioning that, with acetate as the sole carbon source, the production of other metabolites, such as ethanol, succinate, and lactate, was never observed, differently from previous observations when glucose was utilized.

In summary, the strain choice turned out as the decisive factor positively influencing diols production from acetate, with *E. coli* W_pET_*budBAC* producing 10.24-fold higher diols titer compared to its relative control, *E. coli* BL21 (DE3)_pET_*budBAC* (Figure 2B). Therefore, *E. coli* W_pET_*budBAC* (named W-BDO hereafter) was selected for the subsequent acetate uptake and utilization optimization experiments.

### 2.2 Optimization of the Acetate Uptake and Utilization Pathways of the Diols-Producing *E*. *coli* Strain through Gene Overexpression

With the baseline diols-producing *E. coli* W-BDO strain in hand, we asked whether diols production could benefit from improved acetate activation and utilization. In *E. coli* two acetate uptake pathways, mediated by AckA-Pta or Acs, lead to the formation of acetyl-CoA that can enter the TCA cycle (Figure 1). Acetyl-CoA condenses with oxaloacetate to form citrate, which is subsequently oxidized to regenerate the oxaloacetate, with concomitant evolution of two molecules of CO_2_ for each molecule of acetate entering the cycle.^63^ When cells are grown on acetate, which is a C_2_ substrate, the flux toward the glyoxylate shunt increases to preserve the metabolized carbon from its dissipation in CO_2_ in the two decarboxylation reactions of the TCA cycle and to allow the formation of C_4_ precursors for amino acid biosynthesis or gluconeogenesis.^16,64,65^ In the glyoxylate shunt, a part of the carbon flux is diverted at the level of isocitrate, which is derived from acetyl-CoA through citrate, and converted into malate by the combined activity of the isocitrate lyase (AceA) and of the malate synthase (GlcB). Furthermore, malate can be converted into pyruvate, which is the precursor required for diols production, by the malate dehydrogenase enzyme, whose encoding gene is present in two copies in the genome of *E. coli*, *maeA* and *maeB*. The corresponding enzymes differ in their structural and kinetic properties, in their cofactors (with MaeA active with NAD+ or NADP+ and MaeB only with NADP+), in the reversibility of the reaction (malate oxidative decarboxylation or reductive pyruvate carboxylation), which is more marked for MaeA than for MaeB, but also in the regulatory aspects, with MaeB being the most subject to metabolic control.^66^ In particular, acetyl-phosphate was found to increase the activity of MaeB in the direction of the oxidative decarboxylation of malate, while acetyl-CoA was found to inhibit MaeB and did not produce any effect on MaeA.^66^ Therefore, we constructed six additional *E. coli* strains to analyze the effect of the individual or combined overexpression of the *acs*, *ackA-pta*, *aceA*, *maeA*, and *maeB* genes on the diols biosynthetic capacity.

#### 2.2.1 Coordinated Overexpression of the Genes Responsible for the Acetate Uptake and Malate–Pyruvate Conversion Pathways Improves Diols Production in Acetate Fed-Batch Configuration

The *acs*, *aceA*, *glcB*, and *maeA* genes were expressed into pCDFDuet1 and transformed into the *E. coli* W-BDO strain. The acetate consumption and diols production of the resulting strain, *E. coli* W_pET_*budB*-*budA*-*budC* – pCDF_*acs*-*aceA*-*glcB*-*maeA*, hereafter named W-BDO-AC (Table 4), were investigated using either acetate batch or acetate fed-batch configuration in shake flask experiments.

##### 2.2.1.1 Batch Configuration

**Figure 3:**
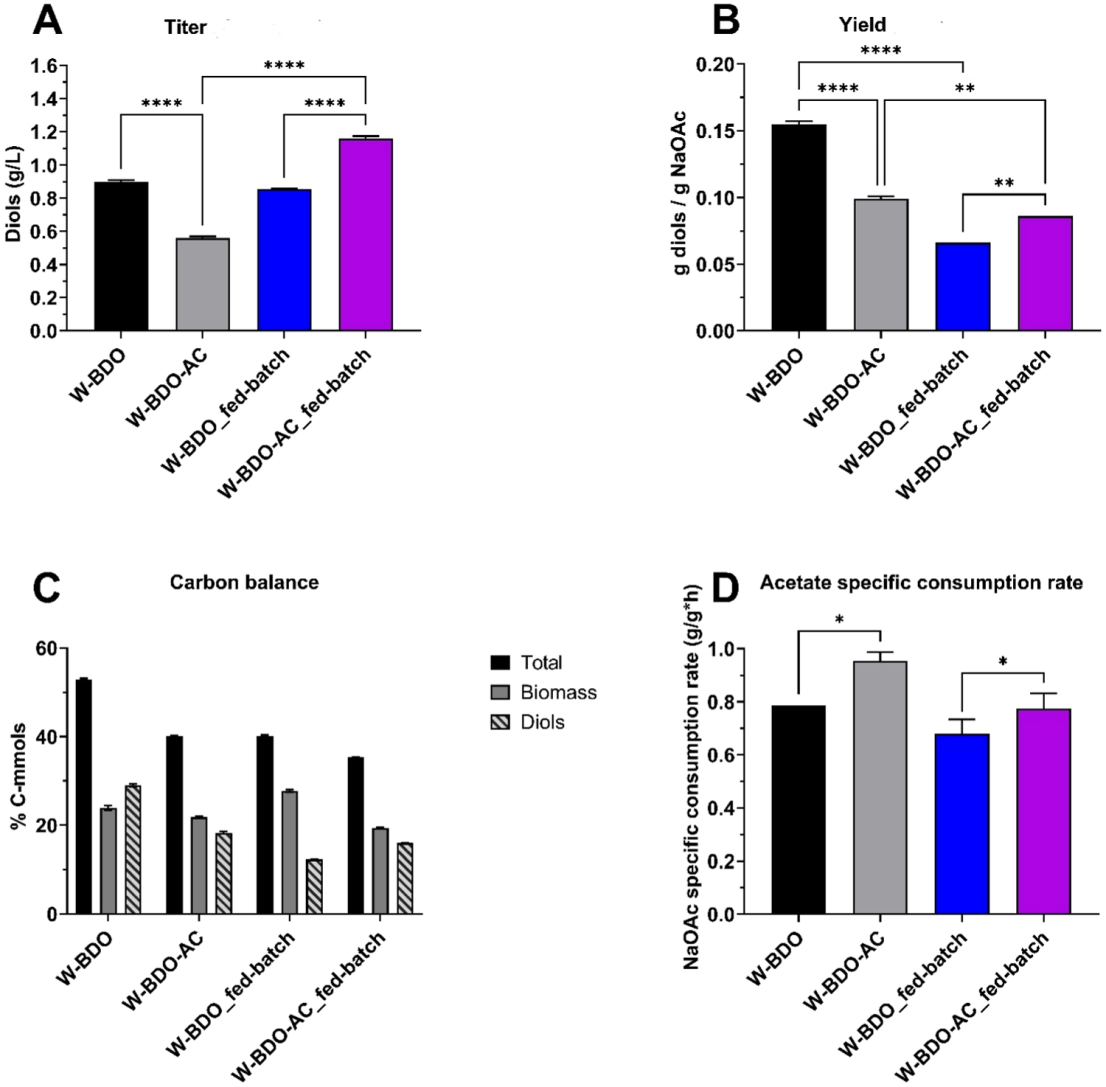
Total diols concentrations, carbon balance analysis, maximum diols yields, and specific acetate consumption rate of the *E. coli* W-BDO and *E. coli* W-BDO-AC strains, cultivated in either acetate batch or fed-batch configurations. *E. coli* W-BDO (W_pET_budB-budA-budC) either in batch (black) or fed-batch (blue) configurations, and *E. coli* W-BDO-AC (W_pET_budB-budA-budC – pCDF_acs-aceA-glcB-maeA) either in batch (gray) or fed-batch (purple) configurations. (A) Total diols (2,3-BDO and acetoin) maximum titers. (B) Diols maximum yields (g/g). (C) Carbon balance analysis as a molar percentage of the supplied carbon in the products (details in Table 5). (D) Maximum acetate specific consumption rate (g/g_CDW_/h). The mean of two independent replicates is plotted for each tested condition. Error bars indicate the standard error of the mean (SEM). Tukey’s test P: **P < 0.0021 and ****P < 0.0001.

The overexpression approach increased acetate uptake as the engineered *E. coli* strain W-BDO-AC consumed sodium acetate at a higher specific consumption rate (0.93 ± 0.06 g/g_CDW_/h) compared to the control strain W-BDO (0.79 ± 0.00 g/g_CDW_/h), as shown in Figure 3. Consequently, W-BDO-AC grew at a 2.09-fold higher rate compared to the control strain W-BDO, as shown in Figure S2. However, the intervention failed to increase diols production. Indeed, the diols titer for W-BDO was 1.61-fold higher (0.90 ± 0.01 g/L) than W-BDO-AC (0.56 ± 0.01 g/L). Consequently, a higher diols yield was obtained by W-BDO (0.16 ± 0.00 g/g) compared to W-BDO-AC (0.10 ± 0.00 g/g) (Figure 3). One positive aspect influenced by the tested overexpression is a tiny increase in volumetric productivity (0.08 ± 0.00 g/L/h for W-BDO-AC compared to 0.05 ± 0.00 g/L/h for W-BDO).

##### 2.2.1.2 Fed-batch configuration

*E. coli* W-BDO and W-BDO-AC were tested also in acetate fed-batch configuration by supplying 9 g/L of sodium acetate to the initial 5 g/L at four time points of the fermentation test, after 6.5, 11, 24, and 30 h (Figure S3). The acetate specific consumption rate of W-BDO-AC (0.78 ± 0.06 g/g_CDW_/h) increased over that of the control W-BDO (0.68 ± 0.07 g/g_CDW_/h), as shown in Figure 3D, albeit at a lower extent when compared to the batch mode. The effect of the intervention on diols production in the fed-batch configuration was the opposite with respect to the batch one. Indeed, W-BDO-AC achieved a higher diols titer (1.16 ± 0.01 g/L) compared to W-BDO (0.85 ± 0.00 g/L), and a slightly higher diols yield (0.09 ± 0.00 g/g) compared to W-BDO (0.07 ± 0.00 g/g) (Figure 3A,B). The volumetric productivities were similar (0.06 ± 0.00 g/L/h for W-BDO-AC compared to 0.05 ± 0.00 g/L/h for W-BDO). W-BDO-AC was preferable to W-BDO also when we considered the carbon flux toward (Figure 3C).

In short, as expected, *E. coli* W-BDO-AC featured improved the acetate specific consumption rate when compared to the *E. coli* W-BDO, thanks to the overexpression of the acetate assimilation pathway. However, this improvement was found to depend on the feeding approach adopted during fermentation. In practice, as for diols yields and titers, the fed-batch fermentation proved preferable to the batch one. This difference may be attributable to the fact that the fed-batch configuration avoids the presence in the culture medium of an excess of acetate, which can produce inhibitory effects on cell growth. Of note, during the fed-batch fermentation W-BDO-AC consumed acetate at a lower rate and achieved a 3-fold higher biomass concentration than during the batch fermentation (Figures S2 and S3). Furthermore, the high biomass concentration afforded by the fed-batch configuration can favor the accumulation of diols, which are nongrowth-associated products, in the stationary phase.

#### 2.2.2 Selective Overexpression of the Genes Involved in the Acetate Uptake and Utilization Pathways

Since the acetate uptake and glyoxylate shunt use alternative pathways (*acs-* or *acK-pta*-mediated) or gene homologues (*maeA*, *maeB*), we explored the effects of intervening on specific enzymes engaged in these pathways, either individually or in combination using the *E. coli* W-BDO strain. To discern the efficacy of the two acetate uptake pathways, either the *acs* gene or the *ackA-pta* genes were separately overexpressed in *E. coli* W-BDO, resulting in the *E. coli* W-BDO-*acs* (W-BDO-AC1) and W-BDO-*ackA-pta* (W-BDO-AC2) strains. Moreover, to verify which form of the malate dehydrogenase enzyme (*maeA* or *maeB*) is more active toward malate conversion into pyruvate, *E. coli* W-BDO-*acs*-*maeA* (W-BDO-AC3), W-BDO-*ackA*-*pta*-*maeA* (W-BDO-AC4), W-BDO-*acs*-*maeB* (W-BDO-AC5) and W-BDO-*ackA*-*pta*-*maeB* (W-BDO-AC5) strains were also developed. Consequently, six new *E. coli* W strains were created as shown in Table 2.

**Table 2:** List of Strains, with Description, Developed during the Overexpression of the Entire Acetate Uptake and Utilization Pathways and Testing in either Batch or Fed-Batch Configurations^a^

strain name	description
W-BDO-AC1	*E. coli* W-BDO overexpressing *acs*. Full name: *E. coli* W_pET_*budB*-*budA*-*budC* – pCDF_*acs*.
W-BDO-AC2	*E. coli* W-BDO overexpressing *ackA* and *pta*. Full name: *E. coli* W_pET_*budB*-*budA*-*budC* – pCDF_*ackA*-*pta*.
W-BDO-AC3	*E. coli* W-BDO overexpressing *acs* and *maeA*. Full name: *E. coli* W_pET_*budB*-*budA*-*budC* – pCDF_*acs*-*maeA*.
W-BDO-AC4	*E. coli* W-BDO overexpressing *ackA, pta* and *maeA*. Full name: *E. coli* W_pET_*budB*-*budA*-*budC* – pCDF_*ackA*-*pta*-*maeA*.
W-BDO-AC5	*E. coli* W-BDO overexpressing *acs* and *maeB*. Full name: *E. coli* W_pET_*budB*-*budA*-*budC* – pCDF_*acs*-*maeB*
W-BDO-AC6	*E. coli* W-BDO overexpressing *ackA*, *pta* and *maeB*. Full name: *E. coli* W_pET_*budB*-*budA*-*budC* – pCDF_*ackA*-*pta*-*maeB*.

a It is worth noting that the pET and pCDF plasmids used were a modified version of the original plasmids. Specifically, the T7 promoter was replaced by the Tac promoter.

These six new *E. coli* W strains were then tested in acetate batch configuration, during flask-based experiments, in terms of cell growth, acetate uptake, and diols production.

##### 2.2.2.1 Cell Growth

Overexpressing *ackA*-*pta* worsened the growth profile compared to overexpressing *acs* (Figure S4). Two of the three strains where the *ackA* and *pta* genes were overexpressed, *E. coli* W-BDO-AC2 and *E. coli* W-BDO-AC6, showed significantly reduced growth rates (0.06 ± 0.00 and 0.04 ± 0.02 ± 0.00 g_CDW_/L/h, respectively), in accordance with previous studies.^67,68^ The negative impact on cell growth caused by the *ack-pta* overexpression can be attributed to the role that can be played by acetyl-phosphate, a signaling metabolite that, by transferring phosphate groups to regulatory proteins, can modulate many processes.^69^ Acetyl-phosphate is the intermediate of the AckA-Pta pathway. Therefore, it is tempting to hypothesize that the overexpression of the genes encoding this pathway can perturb the intracellular levels of acetyl-phosphate, which can eventually result in worsened cell growth.^70^ The strains overexpressing *maeA* (W-BDO-AC3 and W-BDO-AC4) improved the cell growth rate over the strains overexpressing either *acs or ack-pta*, whereas the overexpression of *maeB* improved the growth rate only when it occurred concomitantly with the *acs* but not the *ack-pta* overexpression, as shown in Figure S4. A possible explanation for the MaeB behavior is the fact that the overexpression of *ackA-pta* pathway can cause variations in the acetyl-phosphate pool, which in turn negatively affects the MaeB activity. Indeed, differently from MaeA, MaeB activity is known to be increased by acetyl-phosphate.^66^

##### 2.2.2.2 Acetate Uptake

**Figure 4:**
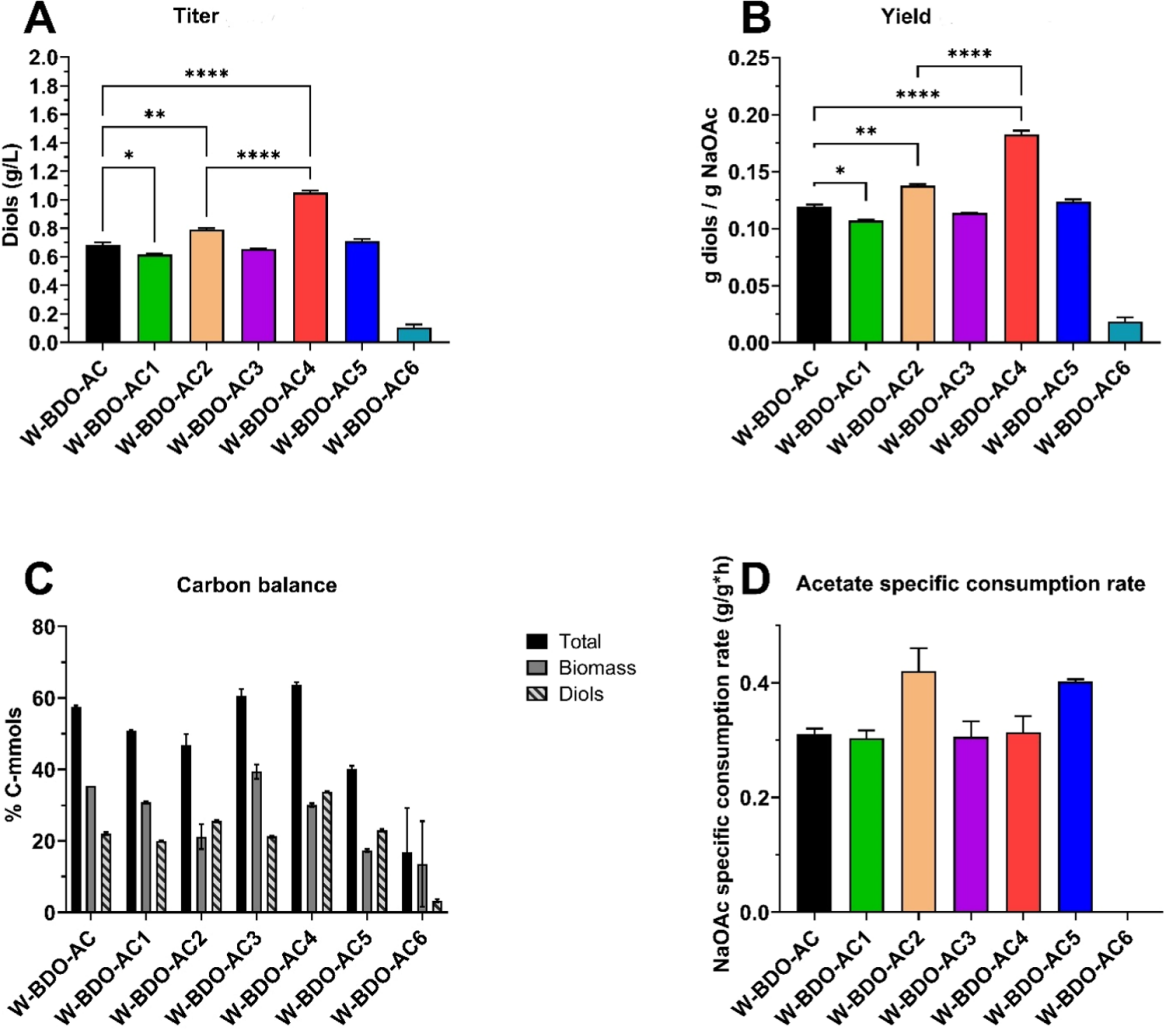
Total diols concentrations, carbon balance analysis, and maximum diols yields of the six different *E. coli* W strains with overexpressed acetate uptake and utilization pathways. The investigated strains *E. coli* W-BDO-AC (W_pET_budB-budA-budC – pCDF_acs-aceA-glcB-maeA) (black), *E. coli* W-BDO-AC1 (W_pET_budB-budA-budC – pCDF_acs) (green), *E. coli* W-BDO-AC2 (W_pET_budB-budA-budC – pCDF_ackA-pta) (light orange), *E. coli* W-BDO-AC3 (W_pET_budB-budA-budC – pCDF_acs-maeA) (purple), *E. coli* W-BDO-AC4 (W_pET_budB-budA-budC – pCDF_ackA-pta-maeA) (red), *E. coli* W-BDO-AC5 (W_pET_budB-budA-budC – pCDF_acs-maeB) (blue), and *E. coli* W-BDO-AC3 (W_pET_budB-budA-budC – pCDF_ackA-pta-maeA) (cyan) were cultivated in a chemically defined medium containing 5 g/L of sodium acetate in batch configuration and incubated at 37 °C and 200 rpm, during 125 mL shake flask batch experiments. (A) Total diols (2,3-BDO and acetoin) maximum titers. (B) Diols maximum yields (g/g). (C) Carbon balance analysis as a molar percentage of the supplied carbon in the products (details in Table 5). (D) Maximum acetate specific consumption rate (g/g_CDW_/h). The mean of two independent replicates is plotted for each tested condition. Error bars indicate the standard error of the mean (SEM). Tukey’s test P: *P < 0.0332; **P < 0.0021 and ****P < 0.0001.

According to our results, the *ackA-pta* overexpression is more effective than that of *acs* in enhancing the *E. coli* W acetate specific consumption rate. *E. coli* W-BDO-AC2 featured an acetate specific consumption rate 1.38-fold higher than the one calculated for *E. coli* W-BDO-AC1 (Figure 4D). The advantage conferred by the *ackA-pta* relative to the *acs* overexpression can be due to the lower ATP expenditure when acetate is activated by Ack-Pta instead of Acs.^48,49,51^ Moreover, the thermodynamic control of the AckA-Pta pathway can constitute a chief advantage compared to Acs by affording the instantaneous adaptation of the fluxes of acetate production and acetate utilization according to the sensed changes in acetate concentration.^48^ The malate dehydrogenase overexpression did not significantly impact the acetate specific consumption rate, irrespective of the genes which were overexpressed to improve acetate activation to acetyl-CoA (Figures 4 and S4).

##### 2.2.2.3 Diols Production

The *ackA-pta* overexpression is preferable to the *acs* one for enhancing diols production as the highest titers were obtained by *E. coli* W-BDO-AC2 and *E. coli* W-BDO-AC4, with the W-BDO-AC4 diols titer 1.69-fold higher than the W-BDO-AC2 one (Figure 4A). The advantage conferred by overexpressing ackA-pta instead of acs is likely due to the lower energy demand of the AckA-Pta pathway.^51^ In addition, comparing the diols titers and yields of *E. coli* W-BDO-AC4 (where *ackA-pta-maeA* were overexpressed) with the ones of *E. coli* W-BDO-AC6 (where *ackA-pta-maeB* were overexpressed) suggests that the malate dehydrogenase MaeA is active toward malate conversion into pyruvate, which is the final precursor for diols production (Figure 4A,B). This finding agrees with previous studies showing that MaeA is involved in gluconeogenesis by providing pyruvate,^71^ while MaeB contributes to the generation of NADPH, which is needed for bacterial growth on two carbon substrates.^72^ Furthermore, it is worth noting that acetyl-CoA was found to inhibit MaeB, whereas it did not produce any effect on MaeA.^66^ Notably, *E. coli* W-BDO-AC4 led to the highest diols titer (1.05 ± 0.01 g/L) and yield (0.18 ± 0.00 g/g) obtained in our study or in similar batch fermentations in the literature. Moreover, the diols yield here achieved is 2.03-fold higher than the highest one reported so far in the literature from acetate, which amounts to 0.09 g/g,^34^ even though we note that the diols yield previously reported was obtained within an acetate fed-batch configuration, while we obtained 0.18 g/g of diols in an acetate batch configuration. Finally, supporting the improvement in diols production lent by overexpressing the *ackA-pta* pathway in combination with *maeA*, is the fact that W-BDO-AC4 led to the highest total carbon conversion and carbon conversion to diols factors, which were quantified in 63.67 ± 0.72 and 33.67 ± 0.21%, respectively (Figure 4C).

### 2.3 Optimization of the Acetate Uptake and Utilization Pathways of the Diols-Producing *E. coli* Strains through Gene Downregulation

A further approach to increase the activity of the promising pathways evaluated so far consisted of deleting known negative regulators of the acetate activation routes and of the glyoxylate bypass. The isocitrate lyase repressor (IcIR) decreases the activity of the acetate utilization pathway by transcriptionally downregulating the *aceBAK* operon, which encloses the genes of the glyoxylate shunt.^51,73−75^ The *iclR* gene deletion in *E. coli* was found to improve the growth rate, acetate consumption, and the titer of several compounds such as itaconic acid,^67^ hydroxy-propionic acid (3-HP), and succinate.^76,77^ Interestingly,^67^ showed that *iclR* deletion is more effective in improving itaconic acid production and acetate consumption than *acs* overexpression in engineered *E. coli*. The acetate uptake pathway acting through Acs is known to be functionally inactivated by the peptidyl-lysine N-acetyltransferase Pka,^62,78^ which acetylates an Acs lysine residue using acetyl-CoA as the acetyl donor.^79^ Downregulating the *pka* gene was previously proven to improve acetate assimilation.^61,80^ Moreover, genetic mutations of *pka* were identified in an acetate-adapted strain of *E. coli* with an increased ability to grow on acetate,^53^ confirming that *pka* is a suitable target for gene deletion to increase acetate utilization.

Inspired by the above-mentioned considerations, we deleted the *iclR* and/or *pka* genes from the genome of the previously developed *E. coli* W-BDO and *E. coli* W-BDO-AC strains. Therefore, six additional strains were developed as reported in Table 3.

**Table 3:** List of *E. coli* Strains Developed during the Optimization of the Acetate Uptake and Utilization Pathways of the Diols-Producing Strains through a Downregulation Approach^a^

strain name	description
W-BDO_Δ*iclR*	*E. coli* W-BDO carrying *iclR* deletion.
W-BDO_Δ*pka*	*E. coli* W-BDO carrying *pka* deletion.
W-BDO_Δ*iclR*+Δ*pka*	*E. coli* W-BDO carrying the double deletion of *iclR* and *pka*.
W-BDO-AC_Δ*iclR*	*E. coli* W-BDO-AC carrying *iclR* deletion.
W-BDO-AC_Δ*pka*	*E. coli* W-BDO-AC carrying *pka* deletion.
W-BDO-AC_Δ*iclR*+Δ*pka*	*E. coli* W-BDO-AC carrying the double deletion of *iclR* and *pka*.

a “Δ” stands for deletion of the specified genes. It is worth noting that the pET and pCDF plasmids used were a modified version of the original plasmids. Specifically, the T7 promoter was replaced by the Tac promoter.

As shown in Figure S5, both W-BDO_Δ*iclR* and W-BDO-AC_Δ*iclR* grew at higher specific growth rates, compared with their relative controls in accordance with previous observations^67^ and achieved higher maximum biomass concentration compared to their controls. Indeed, biomass concentrations of 1.14 ± 0.01, 0.88 ± 0.05, 1.06 ± 0.00, and 0.74 ± 0.05 g_CDW_/L were obtained by W-BDO_Δ*iclR*, W-BDO-AC_Δ*iclR*, W-BDO, and W-BDO-AC, respectively. Similar results were also obtained by deleting *pk*a (Figure S5). On the contrary, the double deletion of both *iclR* and *pka* was detrimental to cells growth as both W-BDO_Δ*iclR*+Δ*pka* and W-BDO-AC_Δ*iclR*+Δ*pka* strains reached biomass concentrations and specific growth rates lower than their relative controls (Figure S5).

**Figure 5:**
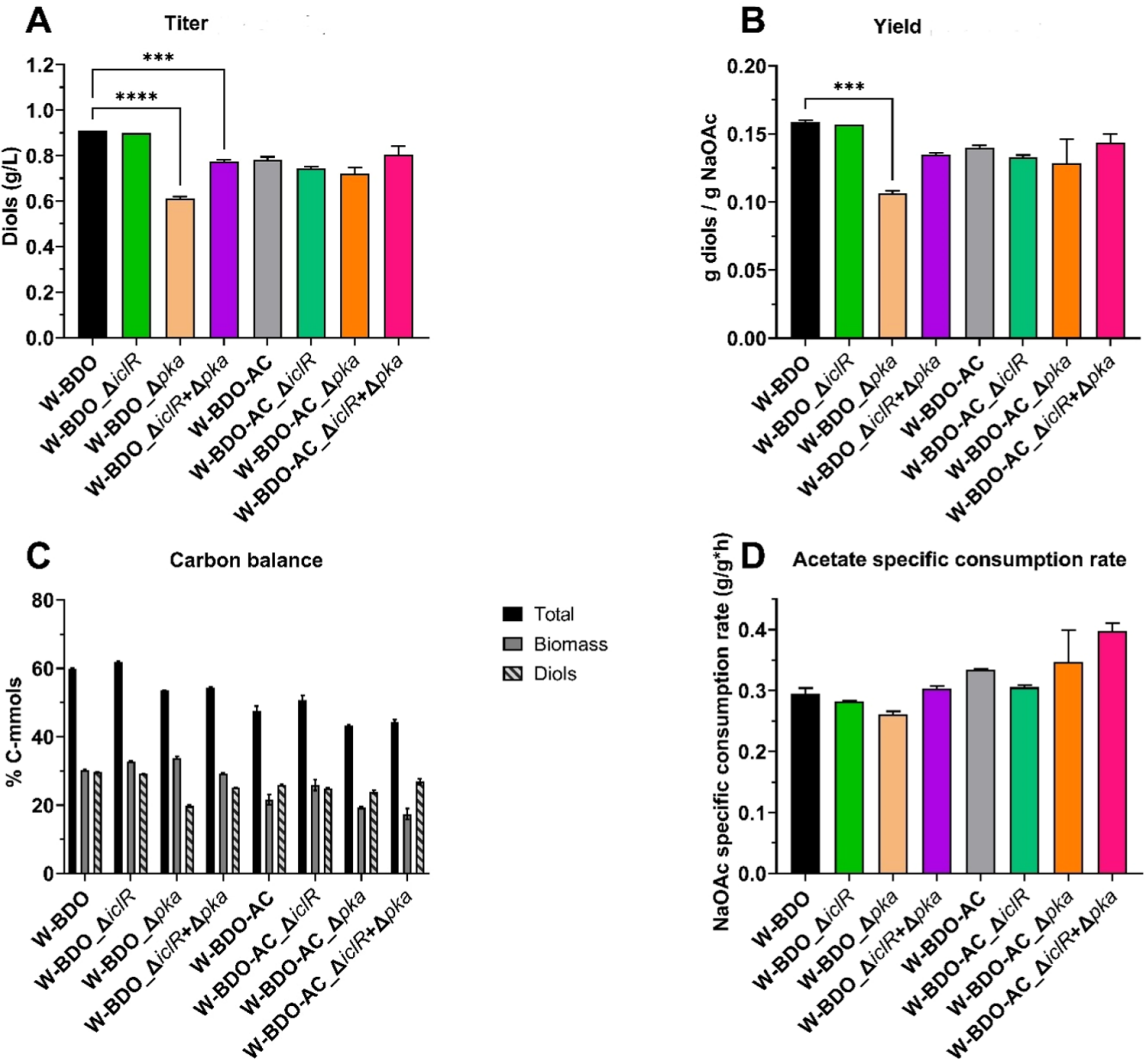
Total diols concentrations, carbon balance analysis, maximum diols yields, and specific acetate consumption rates of the different *E. coli* W strains with deleted iclR and/or pka genes. The investigated strains *E. coli* W-BDO (black), *E. coli* W-BDO_ΔiclR (green), *E. coli* W-BDO_Δpka (light orange), *E. coli* W-BDO_ΔiclR+Δpka (purple), *E. coli* W-BDO-AC (gray), W-BDO-AC_ΔiclR (light green), *E. coli* W-BDO-AC_Δpka (orange), and *E. coli* W-BDO-AC_ΔiclR+Δpka (pink) were cultivated in a chemically defined medium containing 5 g/L of sodium acetate in batch configuration and incubated at 37 °C and 200 rpm, during 125 mL shake flasks batch experiments. (A) Total diols (2,3-BDO and acetoin) maximum titers. (B) Diols maximum yields (g/g). (C) Carbon balance analysis as a molar percentage of the supplied carbon in the products (details in Table 5). (D) Maximum acetate specific consumption rates. (C) Diols maximum yields (g/g). The mean of two independent replicates is plotted for each tested condition. Error bars indicate the standard error of the mean (SEM). Tukey’s test P: ***P < 0.0002 and ****P < 0.0001.

Although individual deletions of the repressors did not statistically significantly improve the acetate consumption rates, it is worthwhile pointing out that W-BDO-AC_ΔiclR+Δpka tangibly increased over the control W-BDO-AC (Figure 5D). Deleting either or both repressor genes, *iclR* and *pka*, did not generally improve diols titer or yield in a statistically significant way (Figure 5A,B). The carbon conversion into diols did not improve because of the downregulation of the individual repressor but the coordinate downregulation of *iclR* and *pka* in W-BDO-AC showed a slight increase over its control. We hypothesize that the *pka* deletion did not achieve the expected effect but rather worsened diols titer and yield, as in the case of W-BDO-AC, since alternative mechanisms can intervene to carry out the function we aimed to repress such as an acetyl-phosphate nonenzymatic acetylation mechanism^81^ or relatively less characterized protein lysine acetyltransferases present in *E. coli*.^82^ On the other hand, it cannot be ruled out that deleting *pka* can change the concentration of acetyl-phosphate that, transferring phosphate groups to regulatory proteins, can affect the functioning of metabolism and finally worsen diols production. Supporting this possible interpretation is the fact that Pka regulates hundreds of acetylation sites particularly present in genes related to translation and central metabolism.^82^ Finally, we can hypothesize that the sole pka deletion, albeit increasing the pool of acetyl-CoA, was not valuable to direct the carbon flux toward the pyruvate-dependent diols biosynthetic pathway. Consistently with this hypothesis, it is worth noting that the coordinate deletion of *pka* and *iclR*, with the latter impinging on the malate-to-pyruvate conversion, at least mildly, improved diols production.

In summary, *E. coli* growth rate and biomass accumulation were slightly enhanced by the single deletion of either *iclR* or *pka*, while the double deletion of *iclR* and *pka* increased the acetate specific consumption rates. On the contrary, the deletion of repressors caused minor effects on diols production.

### 2.4 Acetate Fermentation Scale-Up in Bench-scale Bioreactors

Three *E. coli* strains were selected for scaling up the process in bench-scale bioreactors which enabled a 6-fold increase in the fermentation volume and the pH control: *E. coli* W-BDO, which was selected as the positive control strain since it is able to produce diols, W-BDO-AC, which represents the foundational attempt to promote *E. coli* diol biosynthetic capacity by improving acetate supply and assimilation, and W-BDO-AC_Δ*iclR*+Δ*pka*, which represents an attempt to leverage regulatory features of the acetate uptake and utilization pathways.

**Figure 6:**
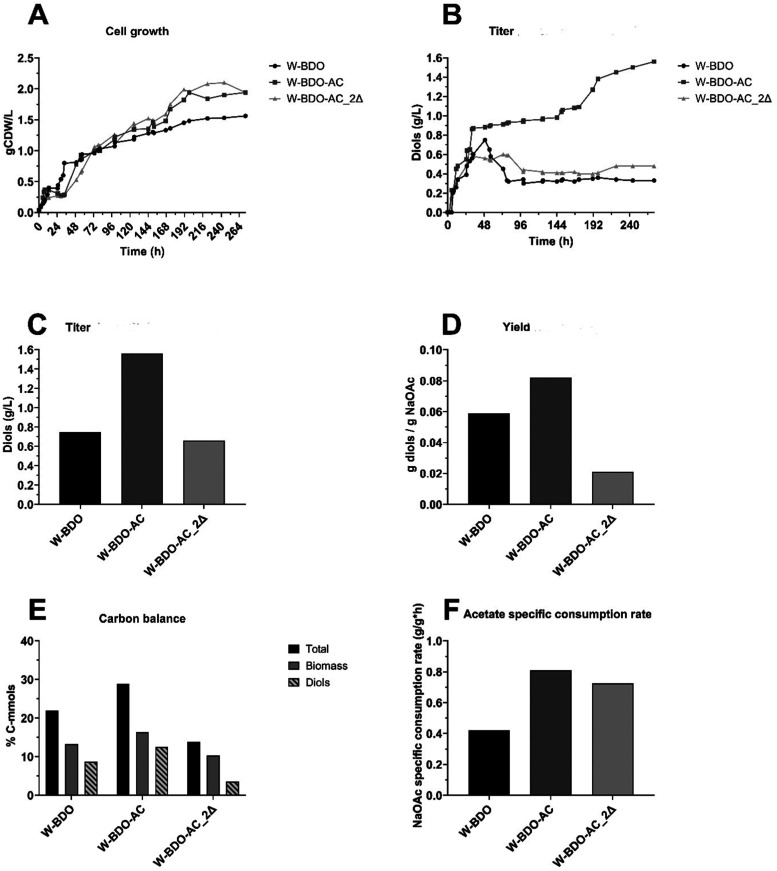
Comparative analysis of growth, diol production, acetate consumption rate, carbon balance analysis, and diols yield of *E. coli* W-BDO, *E. coli* W-BDO-AC, and *E. coli* W-BDO-AC_Δpka+ΔiclR (2Δ) during bioreactor-based experiments. The *E. coli* W-BDO (*E. coli* W_pET_budB-budA-budC) (black), *E. coli* W-BDO-AC (*E. coli* W_pET_budB-budA-budC – pCDF_acs-aceA-glcB-maeA) (dark gray) and *E. coli* W-BDO-AC_ΔiclR+Δpka (2Δ) (*E. coli* W_pET_budB-budA-budC – pCDF_acs-aceA-glcB-maeA – ΔiclR + Δpka) (light gray) strains were cultivated in a chemically defined medium in fed-batch acetate configuration and incubated at 37 °C, 500 rpm and pH between 7.0 and 7.5, during 250 mL bioreactors-based experiments. (A) Growth profiles. (B) Diols production profiles. (C) Total diols (2,3-BDO and acetoin) maximum titers. (D) Total diols maximum yields (g/g). (E) Carbon balance analysis as a molar percentage of the supplied carbon in the products (details in Table 5). (F) Maximum acetate specific consumption rates. This experiment was performed in single replicate.

As shown in Figure 6A, the growth profiles and consequently the specific growth rates of *E. coli* W-BDO, *E. coli* W-BDO-AC, and *E. coli* W-BDO-AC_Δ*pka*+Δ*iclR* strains were similar, suggesting that the metabolic modifications designed did not significantly influence cell growth. Notably, the experiments confirmed that W-BDO-AC improved the acetate uptake rate, which almost doubled with respect to *E. coli* W-BDO (0.42 and 0.81 g/g_CDW_/h for *E. coli* W-BDO and *E. coli* W-BDO-AC, respectively). Conversely, the double deletion of *iclR* and *pka*, conferred a mild negative effect, as shown in Figure 6F.

The overexpression of acs, *aceA*, *glcB*, and *maeA* was beneficial also for diols production from acetate (Figure 6B–D). Indeed, diols titer, yield, and productivity in W-BDO-AC were 2.08-, 1.40-, and 1.32-fold higher than in W-BDO. Accompanying the overexpression of *acs*, *aceA*, *glcB*, and *maeA* with the deletion of *pka* and *icIR*, abolished the advantage gained. Because of the higher biomass and diols titer achieved, W-BDO-AC featured the most promising carbon conversion toward diols as it converted 12.52% of the input carbon into diols and 16.30% in biomass. In comparison, W-BDO converted 21.94% of the input carbon into biomass (13.22%) and diols (8.72%) while W-BDO-AC_Δ*pka*+Δ*iclR* converted 13.77% of the input carbon into biomass (10.29%) and diols (3.48%) (Figure 6E).

In summary, this experiment supports the findings previously discussed. First, W-BDO is a valuable diols-producing *E. coli* strain and can be used as baseline for further metabolic engineering developments. Second, the control of the expression levels of the acetate uptake and malate-to-pyruvate conversion pathway is an effective way to promote diols production, which confirms the potential held by the additional strains constructed in our study. Third, fed-batch cultures operated in pH-controlled environments are confirmed to be valuable ways to ascertain the effects of the metabolic engineering interventions investigated in batch mode in shake flask cultivations. For instance, the last tests confirmed both the substantial inefficacy of W-BDO-AC_Δ*pka*+Δ*iclR* and the superiority of W-BDO-AC compared to W-BDO. Therefore, similar or even more promising strains constructed in our study such as W-BDO-AC4 are worth being tested in the future in fed-batch fermentation setup. Nonetheless, the findings achieved so far are of note. Indeed, the cultivation of *E. coli* W-BDO-AC in acetate fed-batch mode in bioreactors afforded a diols titer of 1.56 g/L which is, to the best of our knowledge, the highest diols titer that has been reported so far for an acetate-grown engineered *E. coli*.^51^ Indeed, even though the diols yields were comparable, the diols titer achieved in our study corresponded to an increase of ∼ 30% compared to the only other effort carried out so far.^34^

## 3 Conclusions

This study engineered the model organism *E. coli* to afford the microbial bioproduction of the industrially relevant compounds acetoin and 2,3-BDO from the renewable carbon source acetate. Since acetate can be synthesized by a large variety of chemical and/or biological ways,^6,83^ the engineered strains suggested by our study are poised to be integrated in the viable realization of multifaceted processes hinged on the intermediate acetate. The *E. coli* strain W-BDO, harboring the biosynthetic pathway supporting diols production, was used as a baseline to enhance the metabolic flux toward diols by controlling the expression levels of genes involved in the acetate uptake and utilization pathways. The *E. coli* strain W-BDO-AC4 (*E. coli* W_pET_*budB*-*budA*-*budC* – pCDF_*ackA*-*pta*-*maeA*) was particularly remarkable as it featured the highest diols titer (1.05 ± 0.01 g/L of diols) and yield (0.183 ± 0.002 g diols/g acetate) in aerobic acetate batch experiments. Finally, selected strains developed in our work were validated in aerobic acetate fed-batch experiments using bench-scale bioreactors. The titer of 1.56 g/L achieved by *E. coli* W-BDO-AC is worth noticing as it is the highest recorded so far in the literature. Moreover, we believe that our study facilitates the selection of suitable candidates such as W-BDO-AC4 for future investigations.

When engineering *E. coli* strains to afford diols synthesis, preventing carbon loss in CO_2_ becomes decisive to render diols biomanufacturing environmentally competitive. However, to make an impact on the carbon balance, the engineered strains deserve further intervention. Indeed, because of the formation of four moles of CO_2_ per mole of 2,3-BDO produced from acetate, the total carbon conversion factor never surpassed 65%. Therefore, further development of the recombinant strains should aim at carbon loss reduction by drawing on previous attempts such as improving CO_2_ recycling capabilities^84−87^ or hydrogen-dependent CO_2_ reduction to formate.^88,89^ Second, achieving increased levels of pyruvate and NADH^90,91^ will open the possibility to overcome the inherent selectivity limits of diols synthesis. Indeed, the increase in pyruvate and NADH availability will lessen the 2,3-BDO conversion into acetoin, which generally occurs in the last stages of acetate fermentation. Therefore, the recombinant strains here developed can be potentially extended in both directions to enable the construction of diol-specific production strains accompanied by low carbon substrate loss in dissipated CO_2_. In sum, we believe that our study will be valuable in bringing metabolic engineering and bioprocess design concepts that use acetate as a carbon source closer to realistic applications.

## 4 Methods

### 4.1 Bacterial Strains and Medium Compositions

All bacterial strains used in this study are listed in Table 4. *E. coli* BL21 (DE3) (New England BioLabs) and *E. coli* W (ATCC 9637) were used for fermentation experiments. *E. coli* DH5-α (New England BioLabs) was used for all of the subcloning steps prior to transformation to the final host. For cell reactivation from glycerol stock, Lysogeny Broth (LB) medium, containing 10 g/L soy peptone, 5 g/L yeast extract, and 10 g/L sodium chloride, was used. 15 g/L of agar was added to the LB medium for plate cultivation. After cell reactivation, all of the fermentation tests were performed using a chemically defined medium adapted from 34 and 67. The chemically defined medium used for *E. coli*-based fermentation experiments consisted (per liter) of potassium phosphate buffer 100 mM (pH 7), MgSO_4_ 0.2442 g, NH_4_Cl 2 g, NaCl 1g, asparagine × H_2_O 1 g, monosodium glutamate × H_2_O 1 g, arginine 0.3 g, lysine × H_2_O 0.5 g, methionine 0.5 g, thiamine × HCl 4.5 mg, riboflavin 0.53 mg, calcium d-pantothenate 6.8 mg, nicotinic acid 7.5 mg, pyridoxine × HCl 1.75 mg, biotin 0.075 mg, and folic acid 0.055 mg. Amino acids and vitamins were added from separate stocks previously filtered. As a source of trace metal elements, 10 mL per liter of the Trace Mineral Supplement MD-TMS (ATCC) was used. Sodium acetate (NaOAc) was added as a carbon source at a final concentration of 5 g/L, unless otherwise specified. Liquid and solid media were supplemented with the specific antibiotics (Table 4), depending on the strain, 50 μg/L spectinomycin, 50 μg/L carbenicillin, or 50 μg/L kanamycin.

**Table 4:** List of All of the Bacterial Strains and Relative Antibiotic Resistances Employed in This Study^a^

name	description	antibiotics resistance
*E. coli* DH5-α	subcloning host	none
*E. coli* BL21 (DE3)	final cloning host and source for *acs*, *aceA*, *glcB*, maeA, *pta*, *ackA*, *maeB*, *pka*, and *iclR*	none
*E. coli* W (ATCC 9637)	final cloning host	none
*E. coli* BL21 (DE3) p5T7_*budCAB*	*E. coli* BL21_p5T7_*budC-budA-budB*	spectinomycin
*E. coli* BL21 (DE3) p5T7_*budBAC*	*E. coli* BL21_p5T7_*budB-budA-budC*	spectinomycin
*E. coli* BL21 (DE3) pET_*budBAC*	*E. coli* BL21_pET_*budB-budA-budC*	carbenicillin
*E. coli* W pET_*budBAC* (W-BDO)	*E. coli* W_pET_*budB-budA-budC*	carbenicillin
*E. coli* W-BDO-AC	*E. coli* W_pET_*budB-budA-budC* – pCDF_*acs-aceA-glcB-maeA*	Spectinomycin, carbenicillin
*E. coli* W-BDO_Δ*pka*	*E. coli* W_pET_*budB-budA-budC_* Δ*pka*	carbenicillin
*E. coli* W-BDO_Δ*iclR*	*E. coli* W_pET_*budB-budA-budC_* Δ*iclR*	carbenicillin
*E. coli* W-BDO_Δ*pka*+Δ*iclR*	*E. coli* W_pET_*budB-budA-budC_* Δ*pka_*Δ*iclR*	carbenicillin
*E. coli* W-BDO-AC_Δ*pka*	*E. coli* W_pET_*budB-budA-budC* – pCDF_*acs-aceA-glcB-maeA_*Δ*pka*	spectinomycin, carbenicillin
*E. coli* W-BDO-AC_Δ*iclR*	*E. coli* W_pET_*budB-budA-budC* – pCDF_*acs-aceA-glcB-maeA_*Δ*iclR*	spectinomycin, carbenicillin
*E. coli* W-BDO-AC_Δ*pka*+Δ*iclR*	*E. coli* W_pET_*budB-budA-budC* – pCDF_*acs-aceA-glcB-maeA_*Δ*pka_*Δ*iclR*	spectinomycin, carbenicillin
*E. coli* W-BDO-AC1	*E. coli* W_pET_*budB-budA-budC* – pCDF_*acs*	spectinomycin, carbenicillin
*E. coli* W-BDO-AC2	*E. coli* W_pET_*budB-budA-budC* – pCDF_*ackA-pta*	spectinomycin, carbenicillin
*E. coli* W-BDO-AC3	*E. coli* W_pET_*budB-budA-budC* – pCDF_*acs-maeA*	spectinomycin, carbenicillin
*E. coli* W-BDO-AC4	*E. coli* W_pET_*budB-budA-budC* – pCDF_*ackA-pta-maeA*	spectinomycin, carbenicillin
*E. coli* W-BDO-AC5	*E. coli* W_pET_*budB-budA-budC* – pCDF_*acs-maeB*	spectinomycin, carbenicillin
*E. coli* W-BDO-AC6	*E. coli* W_pET_*budB-budA-budC* – pCDF_*ackA-pta-maeB*	spectinomycin, carbenicillin

a It is worth noting that the pET and pCDF plasmids used were modified versions of the original plasmids. Specifically, the T7 promoter was replaced with the Tac promoter.

As a procedural note that applies to all experiments, medium formulation was carefully selected to include amino acids and vitamins, that were proven to be beneficial for *E. coli* growth on acetate and for the generation of pyruvate-derived products, such as 2,3-BDO and acetoin.^34,92−94^ Additionally, the initial pH of the acetate fermentation tests was set between 7.0 and 7.5 since an acid environment resulted in a decreased ability of *E. coli* to consume acetate^52^ and a slightly alkaline pH was shown to improve *E. coli* tolerance toward acetate.^95^

### 4.2 Construction of Plasmids and Strains

Gibson Assembly^96,97^ was used for all of the cloning steps performed in this study, with Gibson Assembly Master Mix from New England BioLabs. All primers used in this study were purchased from Sigma Millipore (USA) and are listed in Table S1. The genes *budA*, *budB* and *budC* from *Enterobacter cloacaesubsp. dissolvens* for diols production were synthesized by GeneArt Gene Synthesis (Thermo Fisher Scientific) while the remaining genes of the acetate uptake and utilization pathway, such as *acs*, *aceA*, *glcB*, *maeA*, *pta*, *ackA*, *maeB*, *pka*, and *iclR*, were derived from *E. coli* BL21 (DE3) wild-type (WT). All of the backbones used in this study are listed in Table S2. In particular, the p5T7 backbone was already available in Gregory Stephanopoulos’ laboratory, while pETDuet1, pCDFDuet1, and pCASsac backbones were a kind gift from Xun Wang (PhD visitor in Gregory Stephanopoulos’ laboratory from NJFU). Briefly, plasmids were extracted from the bacterial host using the ZymoPURE Plasmid Miniprep Kit (Zymo Research, USA) and PCR-amplified using specific primers. After the PCR reaction, a 1% agarose gel was run, and the DNA parts were extracted and purified from the gel using the Zymoclean Gel DNA Recovery Kit (Zymo Research). The *budA*, *budB*, and *budC* genes and the genes of the acetate uptake and utilization pathways were also PCR-amplified using specific primers and, after gel extraction, assembled with the backbone parts. The constructed plasmids were transformed by heat shock into *E. coli* DH5-α competent cells (New England BioLabs, USA), following the supplier specifications, and cells were grown overnight in solid LB medium and added with the specific antibiotics. A single colony from the plate was used to inoculate a liquid culture, and the liquid culture was used to extract plasmid DNA using the ZymoPURE Plasmid Miniprep Kit (Zymo Research). The extracted plasmid DNA was transformed into the final host, either by heat shock into *E. coli* BL21 (DE3) (New England BioLabs) following the supplier’s specifications or by electroporation (2500 V, 200 Ω, 2 mm) into *E. coli* W (ATCC 9637). Plasmid and genomic DNA were quantified using a NanoDrop 1000 (Thermo Fisher Scientific), and DNA sequencing was performed after each subcloning step.

Gene deletion using the CRISPR-Cas9 method was performed as previously described (Jiang, 2015). Briefly, competent cells of wild-type *E. coli* W were transformed by electroporation (2500 V, 200 Ω, 2 mm) with a pCAS plasmid and incubated overnight. Subsequently, competent cells of *E. coli* W_pCAS were prepared as described and then transformed by electroporation (2500 V, 200 Ω, 2 mm) with plasmid pTarget_*pka* and/or plasmid pTarget_*iclR* (Table S2) and incubated overnight. 1.64 g/L (10 mM) of Rhamnose was used for pTarget plasmid release, and 5 g/L glucose and 10 g/L sucrose were used for pCAS plasmid release. Strains were then sequenced to verify the absence of the target genes. gRNA regions were designed using the CRISPR gRNA design tool (www.atum.bio).

### 4.3 Competent Cells Preparation

*E. coli* electrocompetent cells were prepared using glycerol/mannitol density step centrifugation, as previously described.^98^

### 4.4 Preparation of Precultures

All strains were kept at −80 °C in 25% (v/v) glycerol. For cultivations, a glycerol stock was used to inoculate a 5 mL LB liquid culture containing the specific antibiotic, and the liquid culture was incubated overnight at 37 °C in a rotary incubator (Sheldon Manufacturing). Cells were centrifuged at 6000 rpm for 7 min (Beckman Coulter) and washed in potassium phosphate solution (100 mM) and distilled water in a ratio of 1:10. The resuspended cells were inoculated into a 125 mL flask containing 25 mL of the above-described chemically defined medium and incubated overnight at 37 °C and 200 rpm (Sheldon Manufacturing). This step was necessary to adapt bacterial cells to the chemically defined medium used for the fermentation experiments. Specific antibiotics were always added to liquid cultures for plasmid maintenance (Table 4). All of the precultures and fermentation tests (both in flasks and bioreactors) started from an initial OD_600 nm_ of 0.1. The bacterial cells of the adapted precultures were centrifuged at 6000 rpm for 7 min and rewashed in potassium phosphate solution (100 mM) and distilled water in a ratio of 1:10. The resuspended cell pellet was then used to inoculate either shake flask- or bioreactor-based fermentation experiments. The optical density (OD_600 nm_) was regularly measured using an Ultrospec 2100 Pro OD-meter (Amersham Biosciences, U.K.).

### 4.5 Strains Testing in Shake Flask-Based Experiments

Screening of different strains and cultivation conditions was performed in duplicate by using 125 mL shake flasks containing 25 mL of the acetate-based chemically defined medium (or the glucose-based chemically defined medium when specified). Flasks were incubated at 37 °C (or 30 °C, when specified) and 200 rpm (or 300 rpm, when specified) in an incubator (Sheldon Manufacturing). Samples were taken regularly for OD_600 nm_ and HPLC measurements. Isopropyl-β-d-1-thiogalactopyranoside (IPTG) 0.1 mM was used for plasmid induction when an optical density at 600 nm between 0.5 and 0.7 was reached.

### 4.6 Aerobic Acetate Fermentation Experiments in Bench-Scale Bioreactors

Bioreactor-based experiments were carried out in 250 mL Applikon MINIBio Lab bioreactors (Applikon Biotechnology, Netherlands). Bioreactors were filled with 150 mL of the acetate-based chemically defined medium and kept at 37 °C and 500 rpm, and the medium was sparged with 0.1 L/min of air. The initial pH of the chemically defined medium was set to 7.0, and, after growth started, the pH of the fermentation was kept below 7.5 using NaOH 4M. Samples were taken regularly to measure the OD_600 nm_. The samples were centrifuged at 10,000 rpm for 5 min (Beckman Coulter), and the supernatant was used for HPLC analysis after filtration. Bioreactor-based experiments were carried out in an acetate fed-batch configuration. A 50 g/L sodium acetate stock solution (neutralized at pH 7) was used in bioreactor-based experiments to intermittently feed sodium acetate inside the bioreactors. In addition, 0.1 mM of Isopropyl-β-d-1-thiogalactopyranoside (IPTG) was added for plasmids induction when an optical density at 600 nm between 0.5 and 0.7 was reached. 20 mL of Antifoam 204 (Sigma) per liter of fermentation was added at the beginning of bioreactor-based experiments.

### 4.7 Calculation of the Main Fermentation Parameters

For each fermentation, a predefined set of fermentation parameters were calculated (Table 5). For cell dry weight (CDW) calculation of *E. coli*, a correlation coefficient of 0.36 g_CDW_/L - OD was used as previously described.^99^ The fermentation parameters calculated in this study included the specific growth rate (g_CDW_/L/h), the yield for total diols (2,3-BDO and acetoin), (either in grams of product per gram of substrate or in Cmoles of product per Cmoles of the substrate), the volumetric productivity of diols (g/L/h), the specific productivity of diols (g/g_CDW_/h), the acetate volumetric consumption rate (g/L/h), the acetate specific consumption rate (g/g_CDW_/h), the carbon conversion to total diols (2,3-BDO and acetoin), and biomass factors (% of Cmmol), and the total carbon conversion factor (% of Cmmol).

**Table 5:** Summary Table of the Main Fermentation Parameters Evaluated in the Experiments Carried Out within This Study^a^

parameter	unit	description
product concentration	g/L	grams of biomass, total diols, or acetate per liter of reaction
product yield	g/g	grams of total diols obtained per gram of initial substrate
product volumetric productivity	g/L/h	grams of total diols obtained per liter of reaction and per hour
product specific productivity	g/g_CDW_/h	grams of diols obtained per gram of biomass and per hour of reaction
specific growth rate	g_CDW_/L/h	grams of cell dry weight obtained per liter of reaction and per hour
total carbon conversion factor	% of Cmmol	total percentage of millimoles of carbon contained in the initial substrate converted into final products
carbon conversion to product factors	% of Cmmol	percentage of millimoles of carbon contained in the initial substrate converted into total diols or biomass
acetate volumetric consumption rate	g/L/h	grams of acetate consumed per liter and per hour of reaction
acetate specific consumption rate	g/g_CDW_/h	grams of acetate consumed per gram of biomass and per hour

a Abbreviation: g, grams; L, liters; h, hours; CDW, cell dry weight; Cmol, moles of carbon; Cmmol, millimoles of carbon.

### 4.8 Analytics

Quantification of acetate, acetoin, meso-2,3-BDO, and ethanol was carried out using an Agilent Infinity II 1260 HPLC system (Agilent) coupled with a refractive index detector (RID) (Agilent 1100 HPLC G1362A) operating at 50 °C. The Aminex HPX-87H column (300 mm × 7.8 mm, Bio-Rad, Hercules) with a precolumn was eluted isocratically with 14 mM H_2_SO_4_ at a flow rate of 0.6 mL/min and an oven temperature of 60 °C. Before HPLC analysis, culture samples were centrifuged for 10 min at 10,000 rpm (Beckman Coulter) and filtered through 0.22 μm filters (Scharlab, Spain).

### 4.9 Data Analysis

Data analysis and plotting were carried out via GraphPad Prism 9.5.0 software (GraphPad Software). All data derived from either triplicate or duplicate experiments are shown as mean values ± the standard error of the mean (SEM). ANOVA and the follow-up Tukey’s test were carried out to compare the means of the distribution of the fermentation parameters among multiple conditions. In the plots of the figures, multiplicity-adjusted Tukey’s test *P*-values are marked in relation to statistically significant comparisons. In addition, single, paired, and one-tailed *t* tests were carried out to compare the means of the distributions of a certain observable obtained under two different conditions.

## Data Availability

No data was used for the research described in the article.

## Supporting Information Available

List of all of the primers used in the acetate to diols section of this study (Table S1); list of all of the plasmids, and their relative antibiotic resistances, used in the acetate to diols section of this study (Table S2); total diols concentrations, carbon balance analysis and maximum diols yields of the *E. coli* BL21 (DE3)_p5T7_*budCAB*, *E. coli* BL21 (DE3)_p5T7_*budBAC*, *E. coli* BL21 (DE3)_pET_*budBAC*, and *E. coli* W_pET_*budBAC* strains developed and cultivated in a chemically defined medium containing 20 g/L of glucose (Figure S1); comparative analysis of growth, diols production, and substrate consumption of the *E. coli* W-BDO and *E. coli* W-BDO-AC strains cultivated in acetate batch configuration (Figure S2); comparative analysis of growth, diols production, and substrate consumption of the *E. coli* W-BDO and *E. coli* W-BDO-AC strains cultivated in acetate fed-batch configuration (Figure S3); comparative analysis of growth, diols production, and substrate consumption of the six different *E. coli* W strains with overexpressed acetate uptake and utilization pathways (Figure S4); and comparative analysis of growth and diols production of the different *E. coli* W strains with deleted *iclR* and/or *pka* genes (Figure S5) (PDF)
