# Supplementary material for: Metabolic Engineering of E. coli for Enhanced Diols Production from Acetate
Source: ACS Synth Biol. 2025 Mar 19;14(4):1204–19. doi: 10.1021/acssynbio.4c00839 (PMC12012870; doi:10.1021/acssynbio.4c00839)
Supplement: Supplementary file 1 — sb4c00839_si_001.pdf [file sb4c00839_si_001.pdf]

Supporting Information

Metabolic engineering of *E. coli* for enhanced diols production from acetate

Luca Ricci*, Xuecong Cen, Yuexuan Zu, Giacomo Antonicelli, Zhen Chen, Debora Fino, Fabrizio C. Pirri, Gregory Stephanopoulos*, Benjamin M. Woolston*, Angela Re*

*Corresponding authors

Luca Ricci: Department of Chemical Engineering, Massachusetts Institute of Technology, Cambridge, MA 02142, USA; Centre for Sustainable Future Technologies, Fondazione Istituto Italiano di Tecnologia, Via Livorno 60, 10144 Turin, Italy; Department of Applied Science and Technology, Politecnico di Torino, Corso Duca degli Abruzzi 24, 10129 Turin, Italy; RINA Consulting S.p.A. Energy Innovation Strategic Centre. Via Antonio Cecchi, 6, Genoa, Italy. Email address: luca.ricci@rina.org

Gregory Stephanopoulos: Department of Chemical Engineering, Massachusetts Institute of Technology, Cambridge, MA 02142, USA. Email address: gregstep@mit.edu

Benjamin M. Woolston: Department of Chemical Engineering, Northeastern University, 360 Huntington Avenue, 223 Cullinane, Boston, MA 02115, USA. Email address: b.woolston@northeastern.edu

Angela Re: Department of Applied Science and Technology, Politecnico di Torino, Corso Duca degli Abruzzi 24, 10129 Turin, Italy. Email address: angela.re@polito.it

Primers for p5T7_*budCAB*	
**Primer name**	**Sequence (5' → 3')**
BB1_fwd	gatcctgtagactagtgtcgacaagcttgc
BB1_rev	acaggtgaaaccagtaaaacaaagcaactagaacatg
BB2_fwd	gttttactggtttcacctgttctattaggtgttacatg
BB2_rev	tatttctacaggggaattgttatccgctc
BduA_fwd	ctttaactagactagaaataattttgtttaactttaagaaggagatatcatatg
BduA_rev	tatttctagtttagttctcgacggagcg
BduB_fwd	cgagaactaaactagaaataattttgtttaactttaagaaggagatatacatatg
BduB_rev	cgacactagtctacaggatctgcgaaagg
BduC_fwd	acaattcccctgtagaaataattttgtttaactttaataaggagatataccatatg
BduC_rev	tatttctagtctagttaaagaccatgccacc

Primers for p5T7_*budBAC*	
**Primer name**	**Sequence (5' → 3')**
22-05-10_BB1_fwd	atggtctttaactaggaattgtgagcggataac
22-05-10_BB1_rev	atagaacaggtgaaaccagtaaaacaaagcaac
22-05-10_BB2_fwd	gctttgttttactggtttcacctgttctattaggtg
22-05-10_BB2_rev	aaaattatttctagtggggaattgttatccgctc
22-05-10_BudA_fwd	tcgcagatcctgtagactagaaataattttgtttaactttaagaagg
22-05-10_BudA_rev	aaaattatttctacattagttctcgacggagcg
22-05-10_BudB_fwd	ggataacaattccccactagaaataattttgtttaactttaag
22-05-10_BudB_rev	aaaattatttctagtctacaggatctgcgaaag
22-05-10_BudC_fwd	tccgtcgagaactaatgtagaaataattttgtttaactttaataag

22-05-10_BudC_rev	atccgctcacaattcctagttaaagaccatgcc

Primers for pET_*budBAC*

**Primer name**	**Sequence (5' → 3')**
nbudB-F1.FOR	cataatgcttaaaagaaggagatatacatgccgccatccgctcc
nbudB-F1.REV	ctgctgtgcatgtatatctccttcttctacaggatctgcgaaaggtgcag
nbudA-F2.FOR	cgcagatcctgtagaagaaggagatatacatgcacagcagcgcatgtgat
nbudA-F2.REV	ccaccttctgcatgtatatctccttcttttagttctcgacggagcggatgg
nbudC-F3.FOR	gtcgagaactaaaagaaggagatatacatgcagaaggtggcgctcg
nbudC-F3.REV	gtaccgacgtcagcgatcgcctagttaaagaccatgccaccatcaatcagc
BDO-Vec.FOR	gtggcatggtctttaactaggcgatcgctgacgtcggtacc
BDO-Vec.REV	ggatggcggcatgtatatctccttcttttaagcattatgcggccgcaagc

Primers for pCDF_*acs-aceA-glcB-maeA*

**Primer name**	**Sequence (5' → 3')**
new-ace-F1.FOR	acgggttttcatgtatatctccttctttatcaggcctacaaaccgttaccg
new-ace-F1.REV	attcgagctcgaagaaggagatatacatgagccaaattcacaaacacacca
new-ace-F2.FOR	gtaggcctgataaagaaggagatatacatgaaaacccgtacacaacaaattg aag
new-ace-F2.REV	tggtttgactcatgtatatctccttcttgcctacagtcagcaacggttg
new-ace-F3.FOR	tttttggttccatgtatatctccttcttccggggctttttgacgctattaa
new-ace-F3.REV	ctgactgtaggcaagaaggagatatacatgagtcaaaccataacccagagc
new-ace-F4.FOR	gggtaccgacgtcagcgatctagcccggtagccttcactacc
new-ace-F4.REV	aagccccggaagaaggagatatacatggaaccaaaaacaaaaaaacagc gt
new-ace-Vec.FOR	tagtgaaggctaccgggctagatcgctgacgtcggtaccc
new-ace-Vec.REV	gaatttggctcatgtatatctccttcttcgagctcgaattcggatcctgg

Primers for pCDF_*acs*	
**Primer name**	**Sequence (5' → 3')**
1-F.FOR	ctagcccggtagccttcactttacgatggcatcgcgatagcc
1-F.REV	gctcgaagaaggagatatacatgagccaaattcacaaacacaccat
1-Vec.FOR	ctatcgcgatgccatcgtaaagtgaaggctaccgggctaga
1-Vec.REV	tgtttgtgaatttggctcatgtatatctcctt

Primers for pCDF_*ackA-pta*	
**Primer name**	**Sequence (5' → 3')**
2-F.FOR	gctcgaagaaggagatatacatgtcgagtaagttagtactggttctgaac
2-F.REV	ctagcccggtagccttcactttactgctgctgtgcagactgaat
2-Vec.FOR	agtctgcacagcagcagtaaagtgaaggctaccgggctaga
2-Vec.REV	agtactaacttactcgacatgtatatctccttcttcgagctcgaattcg

Primers for pCDF_*acs-maeA*	
**Primer name**	**Sequence (5' → 3')**
3-F1.FOR	ttttggttccatgtatatctccttcttttacgatggcatcgcgatagcc
3-F1.REV	gctcgaagaaggagatatacatgagccaaattcacaaacacaccat
3-F2.FOR	ctagcccggtagccttcactttagatggaggtacggcggtagt
3-F2.REV	atgccatcgtaaaagaaggagatatacatggaaccaaaaacaaaaaaacag cgt
3-Vec.FOR	accgccgtacctccatctaaagtgaaggctaccgggctagatc
3-Vec.REV	tgtttgtgaatttggctcatgtatatctcctt

Primers for pCDF_*ackA-pta-maeA*	
**Primer name**	**Sequence (5' → 3')**
4-F1.FOR	gctcgaagaaggagatatacatgtcgagtaagttagtactggttctgaac
4-F1.REV	ttttggttccatgtatatctccttcttttactgctgctgtgcagactgaat

4-F2.FOR	ctagcccggtagccttcactttagatggaggtacggcggtagt
4-F2.REV	gcagcagtaaaagaaggagatatacatggaaccaaaaacaaaaaaacagcgt
4-Vec.FOR	accgccgtacctccatctaaagtgaaggctaccgggctagatc
4-Vec.REV	agtactaacttactcgacatgtatatctccttcttcgagctcgaattcg

Primers for pCDF_*acs-maeB*

Primer name	Sequence (5' → 3')
5-F1.FOR	taactggtcatccatgtatatctccttcttttacgatggcatcgcgatagcc
5-F1.REV	gctcgaagaaggagatatacatgagccaaattcacaaacacaccat
5-F2.FOR	ctagcccggtagccttcactttacagcggttgggtttgcg
5-F2.REV	ccatcgtaaaagaaggagatatacatggatgaccagttaaaacaaagtgcac
5-Vec.FOR	cgcaaacccaaccgctgtaaagtgaaggctaccgggctagatc
5-Vec.REV	tgtttgtgaatttggctcatgtatatctcctt

Primers for pCDF_*ackA-pta-maeB*

Primer name	Sequence (5' → 3')
6-F1.FOR	gctcgaagaaggagatatacatgtcgagtaagttagtactggttctgaac
6-F1.REV	ctggtcatccatgtatatctccttcttttactgctgctgtgcagactgaat
6-F2.FOR	ctagcccggtagccttcactttacagcggttgggtttgcg
6-F2.REV	cagcagtaaaagaaggagatatacatggatgaccagttaaaacaaagtgcac
6-Vec.FOR	cgcaaacccaaccgctgtaaagtgaaggctaccgggctagatc
6-Vec.REV	agtactaacttactcgacatgtatatctccttcttcgagctcgaattcg

Primers for pTarget_*iclR*

Primer name	Sequence (5' → 3')
iclR-F1.FOR	aatactagtgctgttgcatcgtggttagcgttttagagctagaaatagcaagttaaaataaggct

iclR-F1.REV	gtcttacaagctgataagaagaactcgagtagggataacagggtaataga
iclR-F2.FOR	tgttatccctactcgagttcttcttatcagcttgtaagacggacgtgg
iclR-F2.REV	tacagaaaaaggaggctgtcctttttctggcgggcagagg
iclR-F3.FOR	cctctgcccgccagaaaaaggacagcctcctttttctgtatcgtgg
iclR-F3.REV	ttgcttatggagctgcacatagttgatttccgccgacagg
iclR-Vec.FOR	cctgtcggcggaaatcaactatgtgcagctccataagcaaaagg
iclR-Vec.REV	agctctaaaacgctaaccacgatgcaacagcactagtattatacctaggactg agctagctg

Primers for pTarget_*pka*

Primer name	Sequence (5' → 3')
pka-F1.FOR	taatactagtcggcaggaatggatccggcggttttagagctagaaatagcaa gttaaaataaggct
pka-F1.REV	ggtattactgggcttcattggaactcgagtagggataacagggtaataga
pka-F2.FOR	tgttatccctactcgagttccaatgaagcccagtaataccgggc
pka-F2.REV	cgttgggcaagatttagcgttcctcgctgactcataccggt
pka-F3.FOR	ccggtatgagtcagcgaggaacgctaaatcttgcccaacgc
pka-F3.REV	ttgcttatggagctgcacatacgccataaaccggaacatctacg
pka-Vec.FOR	gatgttccggtttatggcgtatgtgcagctccataagcaaaagg
pka-Vec.REV	ctctaaaaccgccggatccattcctgccgactagtattatacctaggactgag ctagct

Table S1: List of all the primers used in the acetate to diols section of this study. Names and 5' → 3' sequences are reported for each primer.

**Plasmid name**	**Description**	**Resistance**
p5T7	empty vector	spectinomycin
pET_Duet1	empty vector	carbenicillin
pCDF_Duet1	empty vector	spectinomycin
p5T7_*budCAB*	p5T7 carrying diols-producing genes	spectinomycin
p5T7_*budBAC*	p5T7 carrying diols-producing genes	spectinomycin
pET_*budBAC*	p5T7 carrying diols-producing genes	carbenicillin
pCDF_*acs-aceA-glcB-maeA*	pCDF carrying the acetate and utilization genes	spectinomycin
pCDF_*acs*	pCDF carrying *acs*	spectinomycin
pCDF_*ackA-pta*	pCDF carrying *ackA-pta*	spectinomycin
pCDF_*acs-maeA*	pCDF carrying *acs-maeA*	spectinomycin
pCDF_ *ackA-pta -maeA*	pCDF carrying *ackA-pta-maeA*	spectinomycin
pCDF_*acs-maeB*	pCDF carrying pCDF_*acs-maeB*	spectinomycin
pCDF_ *ackA-pta-maeB*	pCDF carrying pCDF_ *ackA-pta-maeB*	spectinomycin
pCAS*sac*	pCAS carrying a gene responsible for plasmid release with glucose or sucrose; kanamycin resistance and λ-Red recombinant system	kanamycin
pTarget_*pka*	Specific for *pka* deletion; N20 region; gRNA; Spectinomycin resistance	spectinomycin
pTarget_*iclR*	Specific for *iclR* deletion; N20 region; gRNA; Spectinomycin resistance	spectinomycin

Table S2: List of all the plasmids, and their relative antibiotic resistances, used in the acetate to diols section of this study. General description and antibiotic resistance are reported for each plasmid.

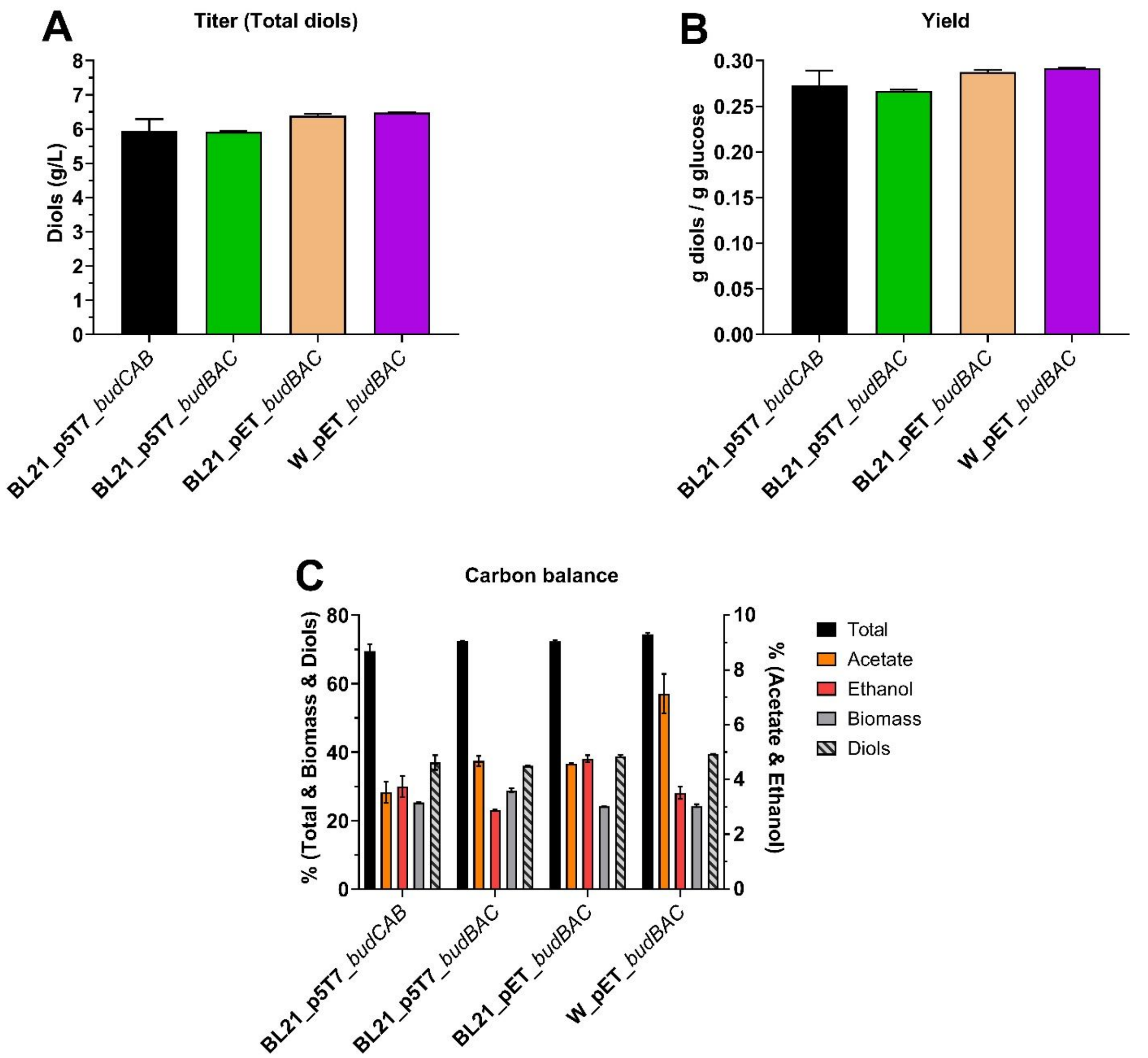

Figure S1. Total diols concentrations, carbon balance analysis and maximum diols yields of the four *E. coli* strains developed and cultivated in a chemically defined medium containing 20 g/L of glucose. The strains were incubated at 37° C and 200 rpm, during 125 mL shake flasks batch experiments. Investigated strains included: *E. coli* BL21 (DE3)_p5T7_*budCAB* (black), *E. coli* BL21 (DE3)_p5T7_*budBAC* (green), *E. coli* BL21 (DE3)_pET_*budBAC* (light orange) and *E. coli* W_pET_*budBAC* (purple). (A) Total diols maximum titers. (B) Diols maximum yields (g/g). (C) Carbon balance analysis. The mean of two independent replicates is plotted for each tested condition. Error bars indicate the standard error of the mean (SEM). No statistically significant differences were found for the four *E. coli* strains investigated.

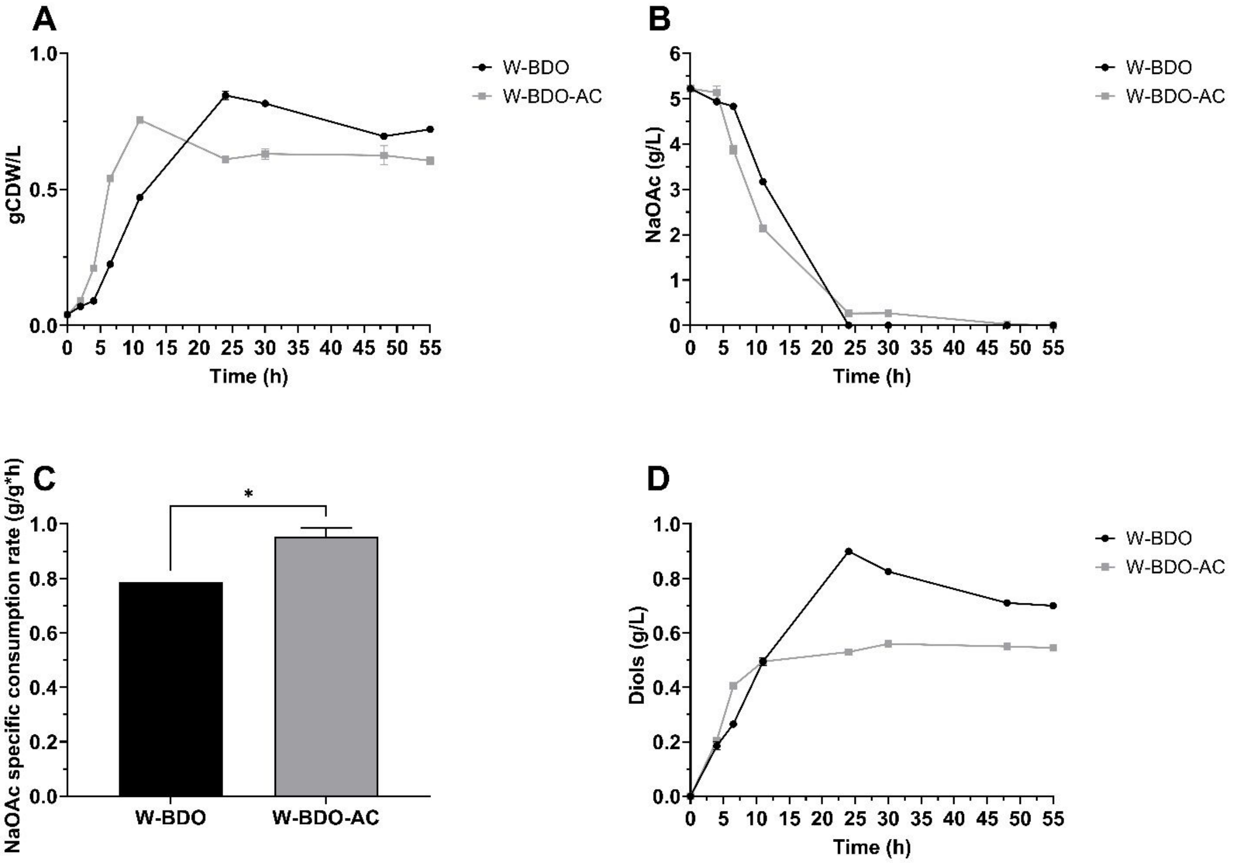

Figure S2. Comparative analysis of growth, diols production and substrate consumption of the *E. coli* W-BDO and *E. coli* W-BDO-AC strains cultivated in acetate batch configuration. *E. coli* W-BDO (W_pET_*budB-budA-budC*) (black) and *E. coli* W-BDO-AC (W_pET_*budB-budA-budC* – pCDF_*acs-aceA-glcB-maeA*) (grey) were cultivated in a chemically defined medium containing 5 g/L of sodium acetate in batch configuration and incubated at 37° C and 200 rpm, during 125 mL shake flasks batch experiments. (A) Growth profiles. (B) Acetate consumption profiles. (C) Maximum acetate specific consumption rates. (D) Diols production profiles. The mean of two independent replicates is plotted for each tested condition. Error bars indicate the standard error of the mean (SEM). Tukey's test P: *P<0.0332.

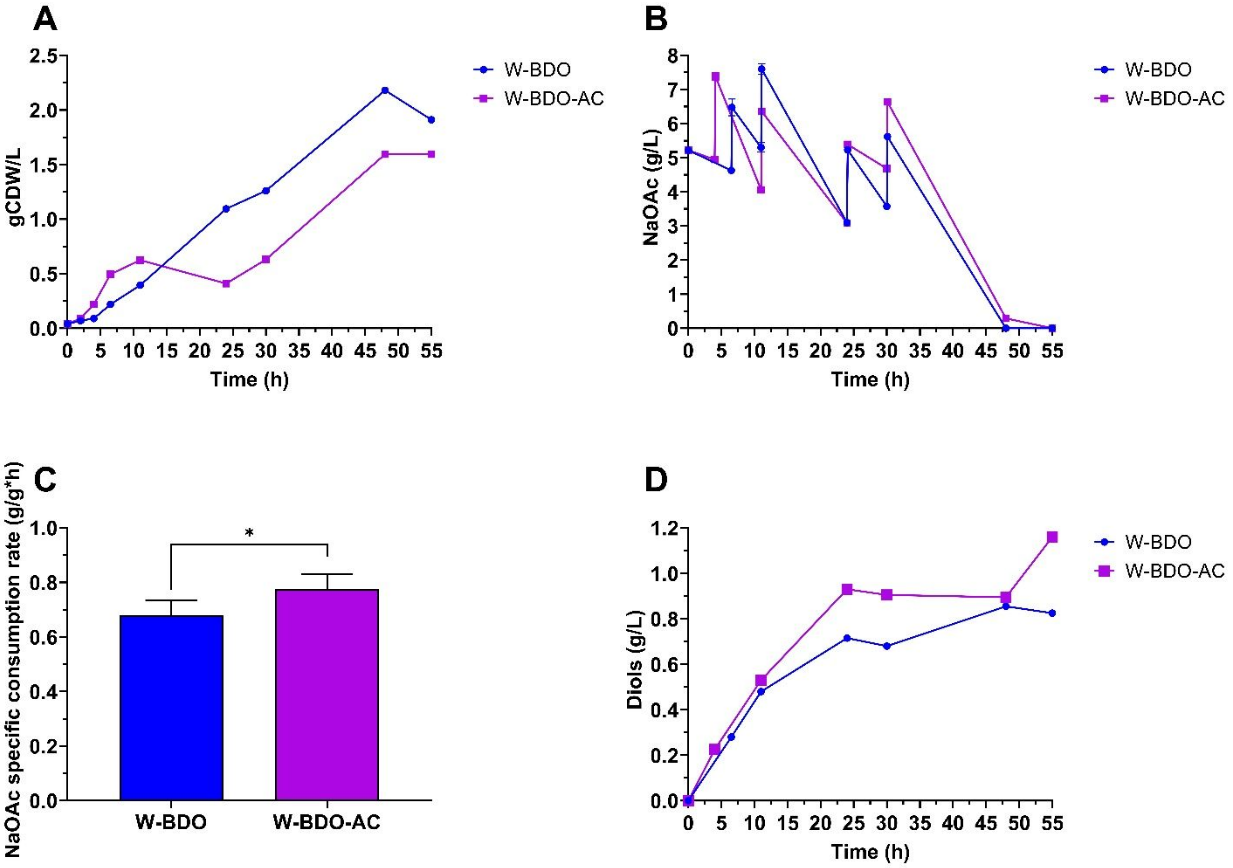

Figure S3. Comparative analysis of growth, diols production and substrate consumption of the *E. coli* W-BDO and *E. coli* W-BDO-AC strains cultivated in acetate fed-batch configuration. *E. coli* W-BDO (W_pET_*budB-budA-budC*) (blue) and *E. coli* W-BDO-AC (W_pET_*budB-budA-budC* – pCDF_*acs-aceA-glcB-maeA*) (purple) were cultivated in a chemically defined medium containing 5 g/L of sodium acetate in fed-batch configuration and incubated at 37° C and 200 rpm, during 125 mL shake flasks batch experiments. (A) Growth profiles. (B) Acetate consumption profiles. (C) Maximum acetate specific consumption rates. (D) Diols production profiles. The mean of two independent replicates is plotted for each tested condition. Error bars indicate the standard error of the mean (SEM). Tukey's test P: *P<0.0332.

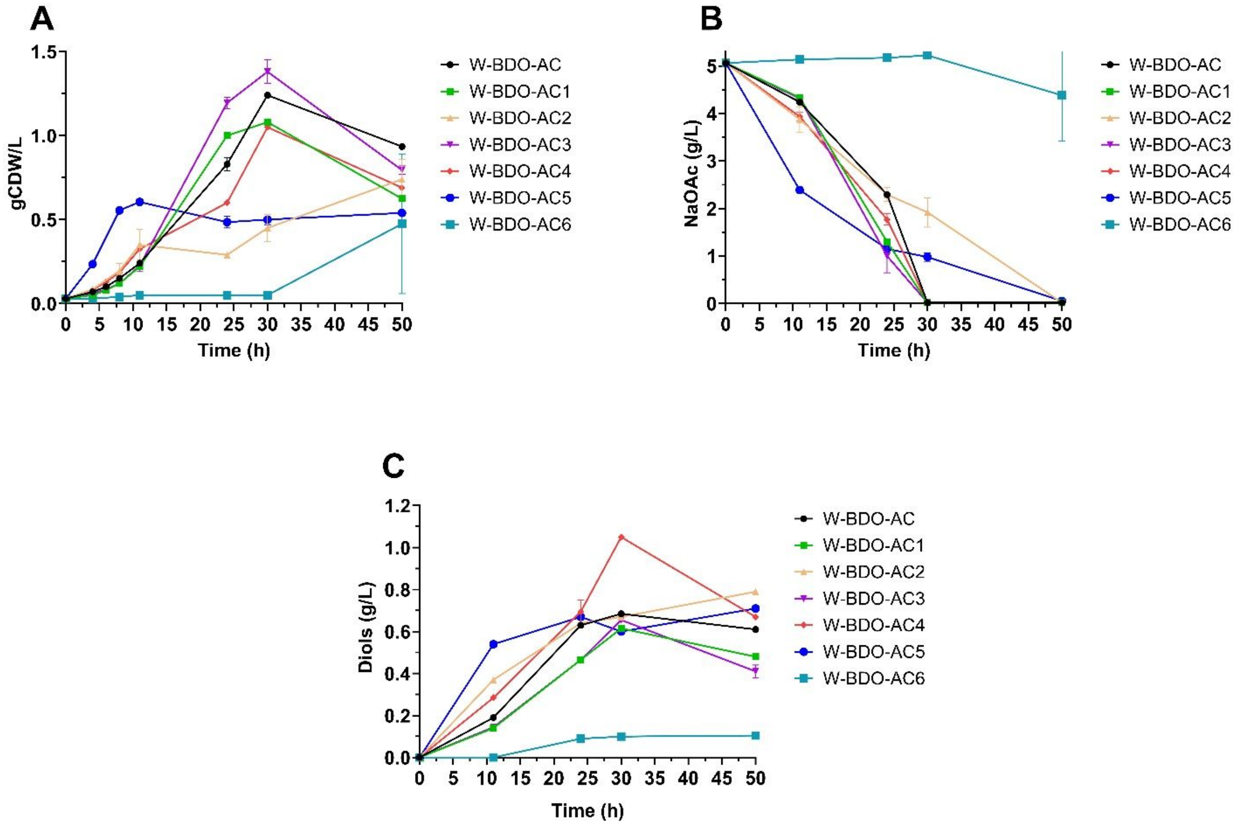

Figure S4. Comparative analysis of growth, diols production and substrate consumption of the six different *E. coli* W strains with overexpressed acetate uptake and utilization pathways. The investigated strains *E. coli* W-BDO-AC (W_pET_*budB-budA-budC* – pCDF_*acs-aceA-glcB-maeA*) (black), *E. coli* W-BDO-AC1 (W_pET_*budB-budA-budC* – pCDF_*acs*) (green), *E. coli* W-BDO-AC2 (W_pET_*budB-budA-budC* – pCDF_*ackA-pta*) (light orange), *E. coli* W-BDO-AC3 (W_pET_*budB-budA-budC* – pCDF_*acs-maeA*) (purple), *E. coli* W-BDO-AC4 (W_pET_*budB-budA-budC* – pCDF_*ackA-pta-maeA*) (red), *E. coli* W-BDO-AC5 (W_pET_*budB-budA-budC* – pCDF_*acs-maeB*) (blue) and *E. coli* W-BDO-AC3 (W_pET_*budB-budA-budC* – pCDF_*ackA-pta-maeA*) (cyan) were cultivated in a chemically defined medium containing 5 g/L of sodium acetate in batch configuration and incubated at 37° C and 200 rpm, during 125 mL shake flasks batch experiments. (A) Growth profiles. (B) Acetate consumption profiles. (C) Diols production profiles. The mean of two independent replicates is plotted for each tested condition. Error bars indicate the standard error of the mean (SEM).

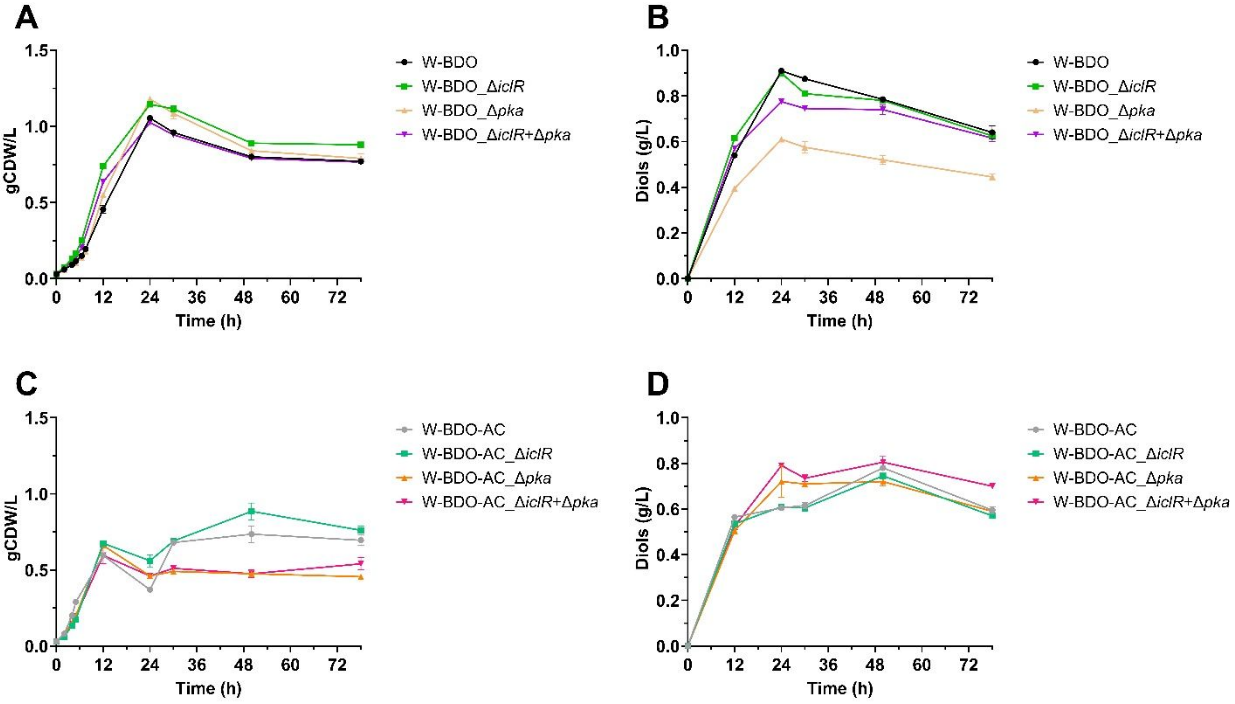

Figure S5. Comparative analysis of growth and diols production of the different *E. coli* W strains with deleted *iclR* and/or *pka* genes. The investigated strains *E. coli* W-BDO (black), *E. coli* W-BDO_Δ*iclR* (green), *E. coli* W-BDO_Δ*pka* (light orange), *E. coli* W-BDO_ΔiclR+Δ*pka* (purple), *E. coli* W-BDO-AC (grey), W-BDO-AC_Δ*iclR* (light green), *E. coli* W-BDO-AC_Δ*pka* (orange) and *E. coli* W-BDO-AC_Δ*iclR*+Δ*pka* (pink) were cultivated in a chemically defined medium containing 5 g/L of sodium acetate in batch configuration and incubated at 37° C and 200 rpm, during 125 mL shake flasks batch experiments. (A) Growth profiles of the W-BDO strains. (B) Diols production profiles of the W-BDO strains. (C) Growth profiles of the W-BDO-AC strains. (D) Diols production profiles of the W-BDO-AC strains. The mean of two independent replicates is plotted for each tested condition. Error bars indicate the standard error of the mean (SEM).
